# Modulation of nongenomic activation of PI3K signalling by tetramerization of N-terminally-cleaved RXRα

**DOI:** 10.1038/ncomms16066

**Published:** 2017-07-17

**Authors:** Liqun Chen, Alexander E. Aleshin, Gulimiran Alitongbieke, Yuqi Zhou, Xindao Zhang, Xiaohong Ye, Mengjie Hu, Gaoang Ren, Ziwen Chen, Yue Ma, Duo Zhang, Shuai Liu, Weiwei Gao, Lijun Cai, Lingjuan Wu, Zhiping Zeng, Fuquan Jiang, Jie Liu, Hu Zhou, Gregory Cadwell, Robert C. Liddington, Ying Su, Xiao-kun Zhang

**Affiliations:** 1 School of Pharmaceutical Sciences, Fujian Provincial Key Laboratory of Innovative Drug Target Research, Xiamen University, Xiamen 361102, China; 2 College of Biological Science and Engineering, Fuzhou University, Fuzhou 350108, China; 3 Sanford Burnham Prebys Medical Discovery Institute, 10901, North Torrey Pines Road, La Jolla, California 92037, USA

## Abstract

Retinoid X receptor-alpha (RXRα) binds to DNA either as homodimers or heterodimers, but it also forms homotetramers whose function is poorly defined. We previously discovered that an N-terminally-cleaved form of RXRα (tRXRα), produced in tumour cells, activates phosphoinositide 3-kinase (PI3K) signalling by binding to the p85α subunit of PI3K and that K-80003, an anti-cancer agent, inhibits this process. Here, we report through crystallographic and biochemical studies that K-80003 binds to and stabilizes tRXRα tetramers via a ‘three-pronged’ combination of canonical and non-canonical mechanisms. K-80003 binding has no effect on tetramerization of RXRα, owing to the head–tail interaction that is absent in tRXRα. We also identify an LxxLL motif in p85α, which binds to the coactivator-binding groove on tRXRα and dissociates from tRXRα upon tRXRα tetramerization. These results identify conformational selection as the mechanism for inhibiting the nongenomic action of tRXRα and provide molecular insights into the development of RXRα cancer therapeutics.

## Abstract (web summary)

The transcription factor retinoid X receptor-alpha (RXRα) can also form homotetramers. Here the authors show that the anti-cancer agent K-80003 selectively inhibits the nongenomic action of N-terminally-cleaved RXRα in tumour cells by stabilizing its tetramerization but not that of full-length RXRα.

Many therapeutic target proteins including nuclear receptors exist in equilibrium between different oligomeric states that control their biological functions^1^,^2^,^3^. Retinoid X receptor α (RXRα), a member of the nuclear receptor superfamily and a validated drug target, exists in different oligomeric forms that regulate a broad spectrum of cellular processes under both physiological and pathophysiological conditions^4^,^5^,^6^,^7^,^8^,^9^,^10^,^11^. Like other nuclear receptors, RXRα comprises an intrinsically disordered N-terminal A/B domain with poorly defined functions, a central DNA-binding domain (DBD), and a ligand-binding domain (LBD) in which α-helices are arranged around a central hydrophobic ligand-binding pocket (LBP)^4^,^5^,^6^,^7^,^8^,^9^,^10^,^11^. Helix 12 (H12), at the C terminus of RXRα, undergoes large movements in response to ligand binding^12^. For example, agonist binding induces H12 to adopt an active conformation that, together with elements of helices H3 and H4, create a groove for binding coactivators that leads to transactivation^10^,^13^,^14^,^15^,^16^. In contrast, in the absence of agonist or in the presence of antagonist, H12 adopts an inactive conformation that favors the binding of corepressors, which serve to inhibit target gene transcription. Coactivators contain interaction domains with an LxxLL motif (NR box), while corepressors utilize a variant of this motif, L/IxxI/VI (co-repressor NR box, CoRNR)^10^,^13^,^14^,^15^,^16^.

In addition to acting as heterodimeric partners of some nuclear receptors^4^,^5^,^6^,^7^,^8^,^9^,^10^,^11^,^17^, RXRα can form homodimers^18^ that trigger specific signalling pathways^19^,^20^,^21^. A unique property of RXRα is an auto-repressive mechanism involving homotetramerization, in which a ‘dimer-of-dimers’ pack in a bottom-to-bottom manner, with H12 from each monomer invading (and invaded by) a neighbouring domain across the tetramer interface^22^. This pairwise ‘exchange of arms’ enables each monomer to bind to and occlude the coregulator-binding groove of its neighbour^22^. Some molecules have been shown to promote the auto-repressed state and repress gene transcription^23^. The equilibrium between inactive homotetramers and active homodimers is tightly regulated by ligand binding^18^,^22^,^24^. Thus, binding of agonists such as 9-*cis*-retinoic acid (9-*cis*-RA) results in dissociation of homotetramers into homodimers, which has been proposed as a regulatory mechanism for RXRα transactivation^22^,^25^. However, compounds that act to promote RXRα homotetramerization remain to be identified and characterized.

Recent advances have revealed important nongenomic functions of RXRα and its non-canonical modulators^7^,^26^,^27^,^28^. We previously found that RXRα is abnormally cleaved in many cancer cells, resulting in a truncated RXRα (tRXRα) that lacks a portion of its N-terminal A/B domain^29^. Unlike the wild-type RXRα, which normally resides in the nucleus, tRXRα is cytoplasmic, and we showed that it interacts with the p85α regulatory subunit of the phosphatidylinositol 3-kinase (PI3K), a critical player in a wide range of cellular processes including cell growth, proliferation, survival, motility, metabolism, protein synthesis and migration^30^,^31^. The interaction with p85α promotes PI3K/AKT activation and enhances tumour cell growth, revealing an oncogenic effect of tRXRα in tumour cells. We also reported that K-80003, a promising anti-cancer agent derived from the non-steroidal anti-inflammatory drug (NSAID) Sulindac, binds to tRXRα and inhibits its interaction with p85α and activation of PI3K signalling^29^. However, the molecular mechanism of this potent inhibition remained to be determined.

Here, we report the crystal structure of RXRα-LBD in complex with K-80003, and the characterization of the role of K-80003-mediated tRXRα tetramerization in regulating its interaction with p85α and the nongenomic activation of PI3K signalling. Our results reveal a previously unrecognized role of RXRα tetramers in modulating subcellular localization and nongenomic interaction with cytoplasmic signalling proteins. Moreover, we show that K-80003 inhibits tRXRα interaction with p85α by stabilizing a tetrameric form of tRXRα through a ‘three-pronged’ mechanism involving both canonical and non-canonical binding. This work opens up new avenues for developing novel RXRα-based therapeutics, by selectively stabilizing a particular oligomeric state.

## Results

### Crystal structure of K-80003-bound RXRα-LBD

**Figure 1:**
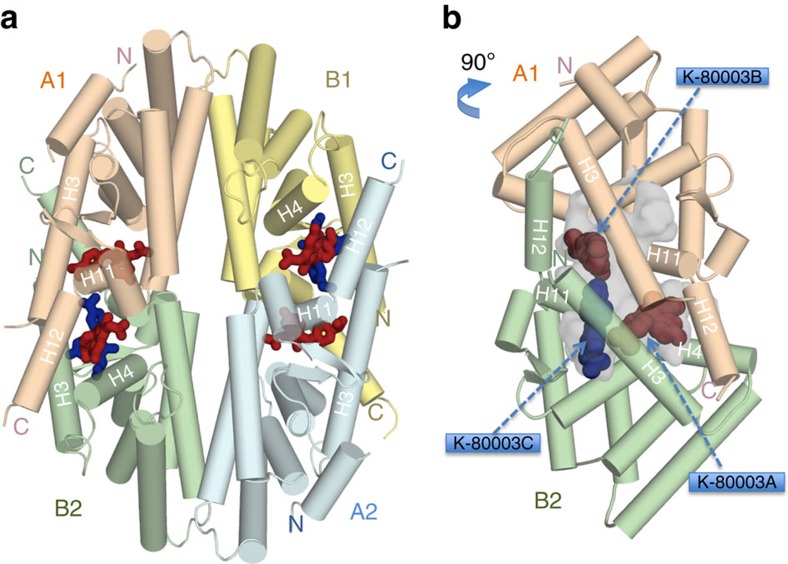
Crystal structure of the RXRα-LBD tetramer in complex with K-80003. (**a**) The RXRα-LBD tetramer in complex with K-80003. The two biological dimers (A1/B1 and A2/B2) are shown as wheat/yellow and green/cyan cartoons, respectively. The bound K-80003 molecules are shown as brown sticks and blue sticks. Helices contributing to the tetramer interfaces are marked. (**b**) The orthogonal view showing the hydrophobic void (semitransparent grey) at the interface between the subunits A1 and B2. The smaller pockets and water channels are removed for clarity. 3 K-80003 molecules (K-80003A, B and K-80003C) filling the cavity are shown as blue and brown balls.

To understand how K-80003 (Supplementary Fig. 1a) modulates the biological activity of tRXRα, we determined the crystal structure of RXRα-LBD in complex with K-80003 to a resolution of 2.6 Å (Table 1). We found that the RXRα-LBD/K-80003 complex adopts a tetrameric structure, similar to those observed in crystals of apo-RXRα or RXRα-LBD/tRA-isomer complex^22^ (Supplementary Fig. 1b), in which two RXRα-LBD canonical homodimers (labelled A1/B1 and A2/B2) pack in a bottom-to-bottom manner (Fig. 1a) with point group symmetry D2. Besides the canonical dimer interface^32^, there are two symmetry-related interfaces involving subunits A1/B2 and B1/A2. These ‘tetramer’ interfaces comprise three sub-regions: parallel packing between symmetry-related H3 helices; ‘end-to-end’ packing between H11 that reduces their length by two helical turns (compared with the agonist-bound structure); and the invasion of each H12 helix into its apposing domain, where it binds to the coregulator-binding groove, consisting of elements of H3 and H4 (Fig. 1a). The apposing domain reciprocates the process in a pairwise ‘exchange of C-terminal arms’. An LMEML motif near the C terminus of H12 binds to the coregulator groove by mimicking the LxxLL/LxxIL motif of coregulators (Supplementary Fig. 2). The tetramer interfaces create two substantial symmetry-related interfacial cavities that are readily accessible to solvent and small molecules.

**Figure 2:**
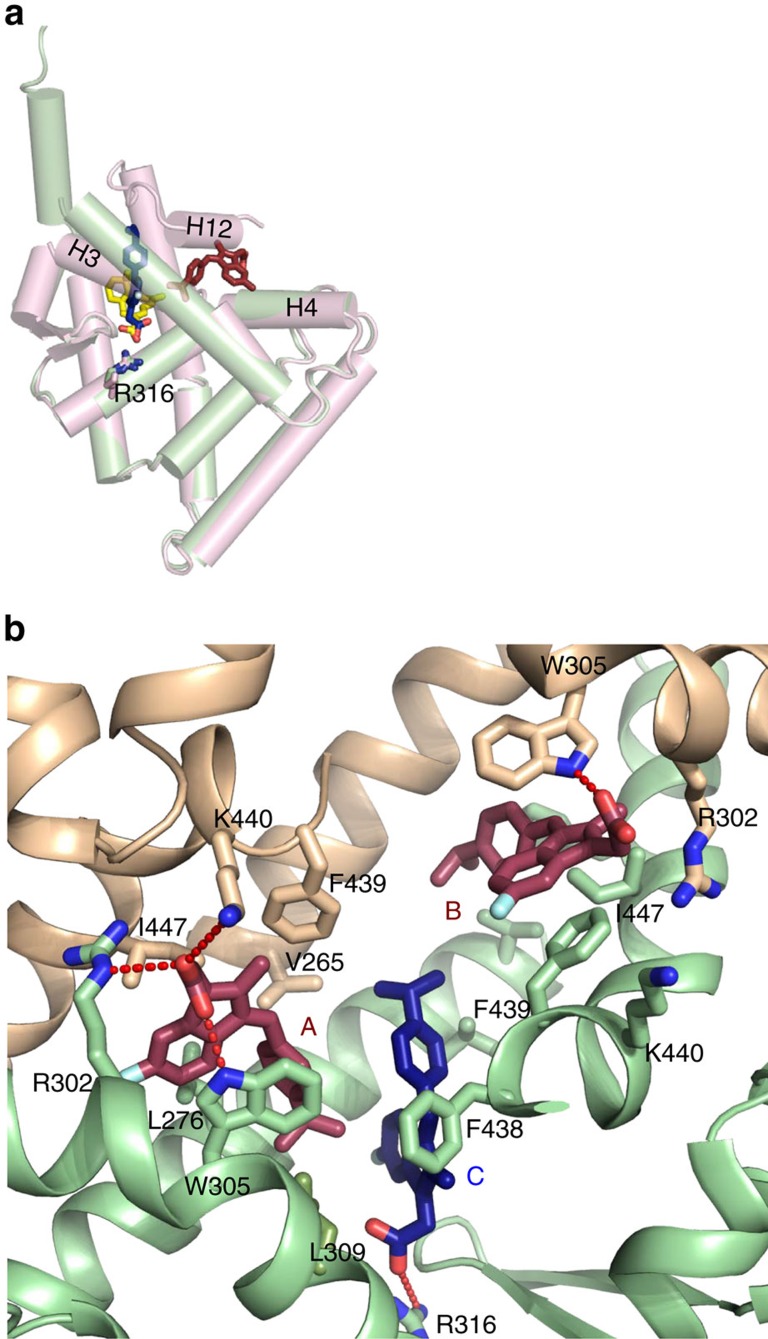
Locations of the bound K-80003 molecules and their interactions with RXRα-LBD. (**a**) Superposition of a monomer subunit (green cartoon) with bound K-80003 molecules K-80003A (brown sticks) and K-80003C (blue/red sticks) on the agonist-binding RXRα-LBD (magenta cartoon, PDB code 1FBY, ligand 9-*cis*-RA in yellow/red stick). (**b**) Binding of the three K-80003 molecules (blue and brown sticks) at the interface between subunits A1 and B2 of the RXRα-LBD tetramer. Selected side-chains of RXRα-LBD subunits that interact with the ligands are shown as sticks.

Refinement of the structure and careful inspection of difference Fourier maps revealed six molecules of K-80003-bound per tetramer, with three molecules per A1/B2 or A2/B1 interfacial cavity (Fig. 1a; Supplementary Fig. 3). This stoichiometry is consistent with values derived by isothermal titration calorimetry (ITC) (Supplementary Fig. 5a). The 2 A1/B2 and A2/B1 interfacial cavities are symmetry-related and the three bound molecules in each cavity (K-80003A, K-80003B and K-80003C, respectively, Fig. 1b) appear to play distinct but complementary roles in stabilizing the tetramer. Thus, K-80003A and K-80003B are arranged about the A1/B2 pseudo-dyad, making similar but distinct interactions (Figs 1b and 2b). They bind in a region where the H12 is located in the agonist-bound RXRα-LBD structure (Fig. 2a) and thus distinct from the canonical ligand-binding region. The indene ring of K-80003A makes many interactions with monomer B2, including parallel aromatic stacking with W305, and hydrophobic binding with L276 and L309, and R302. Its isopropylphenyl ring contacts K-80003C. On the other face of K-80003A, there are several hydrophobic/aromatic interactions, including with the side-chains of F439, I447 and L451 from the A1 monomer (Fig. 2b). On the fourth side, to complete the ‘cage’ around K-80003C, there are numbers of polar and ionic interactions. Notably, the carboxylate of K-80003A makes a bifurcated salt-bridge with K440 from A1 and R302 from B2, as well as an H-bond with the indole N-H of W305 (B2) (Fig. 2b). Thus, K-80003A is firmly encased on all sides at a unique location: by side-chains from A1 and B2 at the top and bottom, by molecule K-80003C on one side, and by elements of the invading H11–H12 turn and H12 helix on the other.

For K-80003B, the symmetry-related packing is similar but distinct with fewer interactions. The ionic interactions with R302 of A1 and K440 of B2 are much weaker, which may explain why K-80003B is less ordered than K-80003A, and with less well-defined electron density. Nevertheless, it makes similar aromatic stacking interactions—in this case, parallel with F439, but a less-optimal (45° stacking angle) with W305 (Fig. 2), as well as hydrophobic interactions with L433 of A1 and L436 of B2.

K-80003C binds in the pocket of monomer B2, in a mode that resembles a canonical ligand (Fig. 2a). Unlike K-80003A and K-80003B, it makes strong contacts only with the B2 subunit (Fig. 2b). Its carboxyl motif makes a salt-bridge with R316, while the indene ring sits in a broad hydrophobic cavity, and both are well-defined in the electron density map. However, the isopropylbenzene moiety sticks out of the pocket and into the cavity, making only weak contacts with other residues; it presumably adopts multiple conformations, consistent with the weak electron density (Supplementary Fig. 3).

Taken together, the interactions between the three bound K-80003 molecules and the protein suggest that K-80003 stabilizes RXRα-LBD tetramer by a unique combination of distinct and canonical-binding mechanisms acting like a ‘3-pronged’ binding mode: K-80003A is tightly packed in a hydrophobic, aromatic and polar cage that strengthens the tetramer and provides additional glue to hold H12 in its place to prevent coregulator binding. K-80003B also contributes to tetramer stabilization, but appears less tightly packed; significantly, however, it blocks the entrance to the unoccupied pocket in monomer A1, which might be otherwise favored by ligands such as 9-*cis*-RA. K-80003C binds in the pocket in monomer B2 interacting with K-80003A and B2.

To understand why K-80003 binds asymmetrically to the tetramer, we closely examined the shape of the tetramer’s interfacial cavity. Superposition of A1/B2 subunit on to itself reveals that A1/B2 is asymmetric (Supplementary Fig. 4a) with a smaller pocket in monomer A1 than in the B2 monomer (Supplementary Fig. 4b). This asymmetry seems to be resulted from the shifts of H3 and H11 into the pocket in A1 (Supplementary Fig. 4c) and provides an explanation for why small molecules bind asymmetrically to the tetramer^22^,^33^. In the previously reported RXRα-LBD/tRA-isomer tetramer structure^22^, tRA-isomer binds to the narrower pocket in monomer A1 where K-80003 molecule is too big to fit in (Supplementary Fig. 4b). In our structure, the large size of the K-80003C molecule presumably selects the wider LBP pocket of the A1/B2 dimer. Despite these differences in ligand binding, the two tetramers have similar overall structures (r.m.s. deviation for Cα atoms is 1.20 Å), and the largest changes are observed at the tetramer interface (Supplementary Fig. 1b), presumably induced by the non-canonical binders, K-80003A and K-80003B.

### K-80003 promotes tetramerization of tRXRα but not RXRα

**Figure 3:**
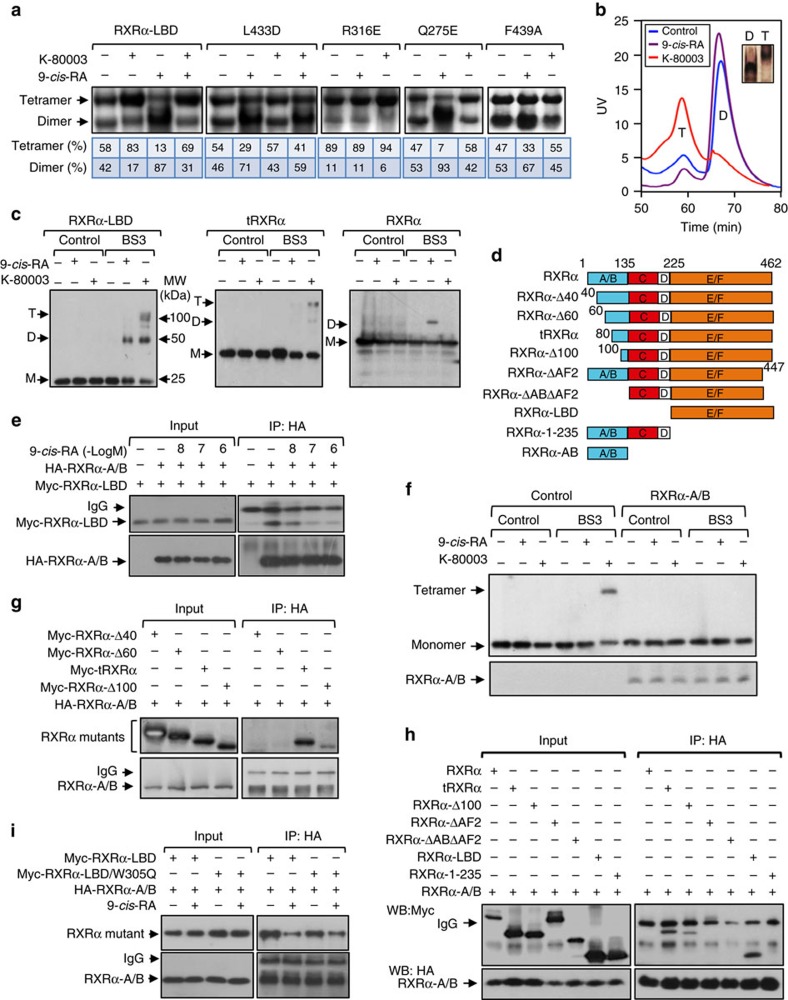
Induction of RXRα tetramerization by K-80003 and its regulation by the N-C intramolecular interaction. (**a**) Equal amounts of purified RXRα-LBD or mutant protein were incubated with DMSO, 9-*cis*-RA, and/or K-80003, and separated by non-denaturing polyacrylamide gel electrophoresis followed by Coomassie Bright Blue staining. The percentage of tetramer and dimer of RXRα-LBD or mutants was quantitated by densitometric analysis of the corresponding blots. One of four similar experiments is shown. (**b**) RXRα-LBD incubated with K-80003 or 9-*cis*-RA was subject to gel filtration chromatogram assay. Results showed that 9-*cis*-RA-induced RXRα-LBD was mostly in dimer (D), while K-80003-induced RXRα-LBD was mostly in tetramer (T). One of three similar experiments is shown. (**c**) HepG2 cells transfected with RXRα, tRXRα or RXRα-LBD were treated with 9-*cis*-RA or K-80003. Cell lysates prepared were then subjected to BS3 crosslinking, and analysed by western blotting using ΔN197 anti-RXRα antibody. One of more than five similar experiments is shown. (**d**) Schematic representations of RXRα and mutants. A/B, C, D, E/F domains in RXRα are indicated. (**e**) HepG2 cells transfected with HA-RXRα-A/B and Myc-RXRα-LBD were treated with 9-*cis*-RA, and analysed by coIP with anti-HA antibody. One of two similar experiments is shown. (**f**) Inhibition of K-80003-induced tRXRα tetramerization by A/B domain. HEK293T cells transfected with tRXRα together with RXRα-A/B were treated with K-80003. Cell lysates were subjected to BS3 crosslinking, and analysed by western blotting using ΔN197 anti-RXRα antibody. One of three similar experiments is shown. (**g**) RXRα-A/B interaction with RXRα N-terminal deletion mutants. HA-RXRα-A/B and Myc-tagged RXRα N-terminal deletion mutants were transfected in to HEK293T cells, and their interaction was analysed by coIP. (**h**) Interaction of RXRα-A/B with RXRα mutants. HA-RXRα-A/B and Myc-tagged RXRα-mutants were transfected together in to HEK293T cells, and their interaction was analysed by coIP. One of three similar experiments is shown. (**i**) Mutation of Trp305 does not affect N/C interaction. Myc-tagged RXRα-LBD or RXRα-LBD/W305Q was transfected together with HA-RXRα-A/B into HEK293T cells in the presence or absence of 9-*cis*-RA (10^−7 ^M). Cell lysates were prepared and analysed by coIP.

The unique binding of K-80003 observed in the tetrameric form of K-80003-bound RXRα-LBD crystal structure prompted us to determine whether K-80003 binding could promote RXRα-LBD tetramerization. In non-denaturing polyacrylamide gel electrophoresis, purified RXRα-LBD protein existed as two distinct bands corresponding to homodimer and homotetramer, respectively (Fig. 3a). As expected, incubation of RXRα with 9-*cis*-RA known to induce homodimerization^18^ resulted in a shift of RXRα-LBD from tetramers to dimers. In contrast, incubation with K-80003 induced an accumulation of tetramers. L433 near the C terminus of H10 packs directly against the indene ring of K-80003B, and its mutation to D would destabilize key interactions with K-80003 due to loss of existing hydrophobic interaction and introduction of repulsive charge–charge interaction with the carboxyl motif of K-80003B. Indeed, K-80003 failed to bind to RXRα-LBD/L433D (Supplementary Fig. 5a) and promote its tetramerization (Fig. 3a). Substitution of Q275 in H3 with E, or F439 in H11 with A, which are involved in the binding of K-80003 (Fig. 2), impaired the tetramerization of respective mutants by K-80003, confirming the role of H3 and H11 in the K-80003-induced stabilization of RXRα-LBD tetramers. Interestingly, mutating R316 in LBP, which is essential for 9-*cis*-RA binding^12^ and involved in K-80003C binding (Fig. 2b), with E, resulted in a mutant (RXRα-LBD/R316E) that exhibited mainly as a tetramer independent of the presence of either 9-*cis*-RA or K-80003, in agreement with a previous report^25^. The effect of K-80003 on stabilizing RXRα-LBD tetramers appeared overpower the 9-*cis*-RA-induced homodimerization as RXRα-LBD was mainly found as a tetramer in the presence of both K-80003 and 9-*cis*-RA (Fig. 3a).

The ability of K-80003 to stabilize RXRα-LBD tetramer was also illustrated by gel filtration chromatography showing that purified RXRα-LBD protein mainly existed as a tetramer in the presence of K-80003, while it displayed predominantly as a homodimeric complex in the presence of 9-*cis*-RA (Fig. 3b). Evaluation of a panel of chemical crosslinkers identified homobifunctional N-hydroxysuccimide-based chemical cross-linker BS3 as the most efficient one to crosslink the RXRα-LBD tetramer (Supplementary Fig. 5b). Thus, BS3 was subsequently used to study the effect of K-80003 on RXRα tetramerization. Extracts from cells transfected with RXRα-LBD and treated with either K-80003 or 9-*cis*-RA were prepared and subsequently incubated with BS3. Figure 3c showed that the treatment of cells with K-80003 produced crosslinked species on SDS-PAGE gels of 25, 50 and 100 kDa, corresponding to monomer, dimer and tetramer of RXRα-LBD, respectively. For comparison, treatment with 9-*cis*-RA resulted in only monomer and homodimer. Interestingly, RXRα or mutants expressed in cells mainly existed as a monomer even after crosslinking, which is different from purified RXRα proteins. Examination of the effect of K-80003 on tetramerization of tRXRα and RXRα revealed that K-80003 effectively promoted the formation of tRXRα tetramers. Unexpectedly, K-80003 failed to promote the tetramerization of the full-length RXRα, indicating that the N-terminal A/B domain interferes with its tetramerization.

### The RXRα N/C interaction inhibits its tetramerization

The above observation prompted us to determine how the N-terminal A/B domain of RXRα inhibited its tetramerization (Fig. 1). Thus, the interaction between RXRα-A/B domain and RXRα-LBD (Fig. 3d) was studied by cell-based coimmunoprecipitation (coIP) assays. Immunoprecipitation of the RXRα-A/B protein resulted in a strong coIP of the RXRα-LBD protein, demonstrating their interaction. The interaction was inhibited by 9-*cis*-RA in a dose-dependent manner (Fig. 3e), and was confirmed by immunostaining showing extensive colocalization of transfected RXRα-A/B with RXRα-LBD but not RXRα in the cytoplasm of cells (Supplementary Fig. 6a). Cotransfection of the RXRα A/B domain completely suppressed the effect of K-80003 on promoting tRXRα tetramerization (Fig. 3f). These data reveal an extensive intramolecular interaction between the N terminus and the C terminus (N/C) in RXRα and its critical role in regulating RXRα tetramerization, and also provides a molecular explanation for the differential effect of K-80003 on the tetramerization of tRXRα and RXRα.

To further study the N/C interaction, we first conducted deletion analysis of the N-terminal A/B region to narrow down the region required for the interaction. Deletion of either the N-terminal 40 or 60 amino acids could not confer the ability of the resulting mutants to interact with the A/B domain (Fig. 3g). Deletion of additional 20 amino acids resulted in a mutant (tRXRα), which strongly interacted with the RXRα-A/B protein. RXRα-Δ60, like tRXRα, interacted strongly with RARγ (Supplementary Fig. 6b), demonstrating that RXRα-Δ60 is still active in heterodimerization with RARγ. These results suggested that amino acids from 60 to 80 are critical for the N/C interaction. We next determined region in RXRα-LBD required for binding A/B domain. RXRα-A/B could interact with RXRα mutants lacking N-terminal sequences (tRXRα, RXRα-Δ100 and RXRα-LBD) but not with mutant lacking C-terminal LBD (RXRα-1-235) or mutants lacking AF2/H12 region (RXRα-ΔAF2 and RXRα-ΔA/BΔAF2) (Fig. 3h). tRXRα without AF2/H12 also failed to interact with RXRα-A/B (Supplementary Fig. 6c). Thus, the N/C intramolecular interaction involves the N-terminal A/B domain and the C-terminal AF2/H12. The conclusion was supported by data showing the inability of the A/B domain to bind to the full-length RXRα, likely due to the unavailability of the C-terminal binding site masked by its own N-terminal A/B domain. The AF2/H12 is involved in the formation of the hydrophobic coactivator-binding groove that was shown to mediate the N/C interaction of some nuclear receptors^34^,^35^. To determine whether the N/C interaction involves the coactivator-binding groove, we tested whether W305, which is located in H5 and was shown to play a critical role in the formation of the coactivator-binding groove^12^, was involved in the N/C interaction. Our results showed that RXRα-LBD/W305Q with W305 mutated to Q, which failed to bind to an LxxLL-containing protein (see below), could still bind to the RXRα-A/B protein in a 9-*cis*-RA sensitive manner similar to the wild-type RXRα-LBD (Fig. 3i). These results preclude the involvement of the coactivator-binding groove in the N/C interaction.

### An LxxLL motif in p85α mediates p85α interaction with RXRα

**Figure 4:**
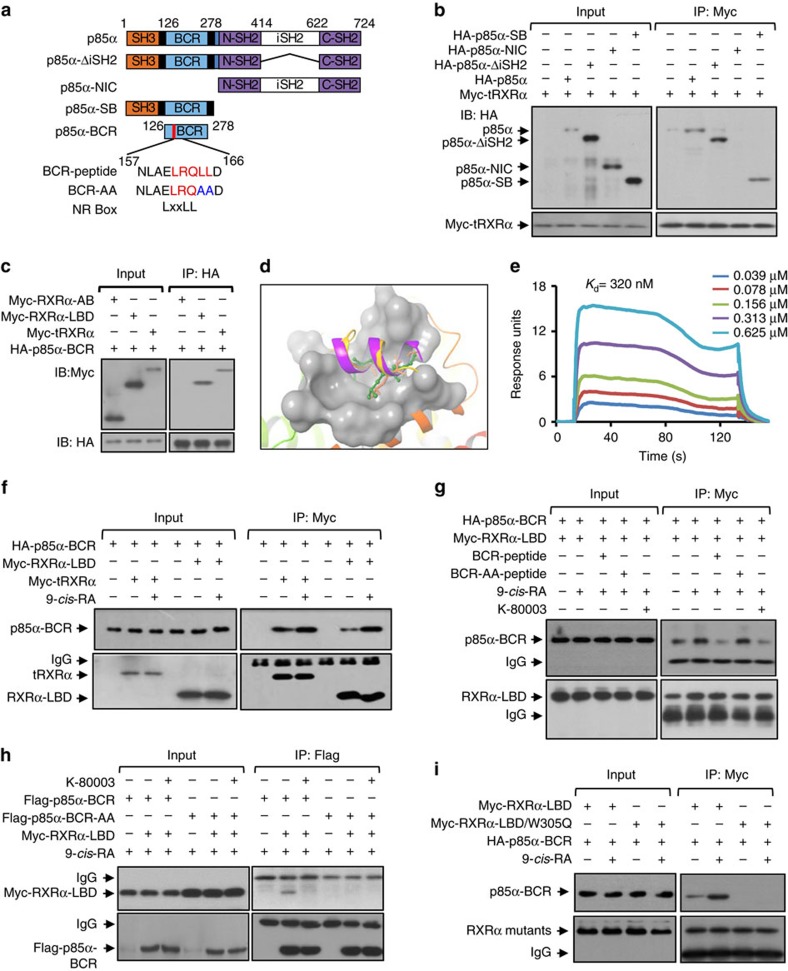
Identification of an LxxLL-like motif in p85α and its interaction with the coactivator-binding groove in tRXRα. (**a**) Schematic representations of p85α and mutants. SH3, BCR, nSH2, iSH2, cSH2 domains in p85α and the location of the BCR-LxxLL motif are indicated. BCR and mutant peptides are shown. (**b**) Interaction of tRXRα with p85α mutants. Myc-tRXRα expression vector was transfected into HEK293T cells along or together with p85α mutant tagged with HA epitope, and their interaction was analysed by coIP using anti-Myc antibody. (**c**) BCR domain interaction with RXRα mutants. HA-p85α-BCR was cotransfected with Myc-tagged RXRα mutants into HEK293T cells, and their interaction was analysed by coIP using anti-HA antibody. (**d**) The BCR-LxxLL peptide (structure was extracted from the BCR crystal structure of PDB code 1PBW) docks to the coactivator-binding groove on RXRα (PDB code 3FUG). (**e**) SPR analysis of BCR-peptide binding to RXRα-LBD in the presence of 9-*cis*-RA. (**f**) Enhancing effect of 9-*cis*-RA on p85α-BCR interaction with RXRα-LBD and tRXRα. Cells transfected with Myc-tRXRα or Myc-RXRα-LBD together with HA-p85α-BCR were treated with 9-*cis*-RA (10^−7 ^M), and analysed by coIP. One of two similar experiments is shown. (**g**) Inhibitory effect of LxxLL-containing BCR peptide on RXRα-LBD interaction with p85α-BCR. HEK293T cells transfected with HA-p85α-BCR and Myc-RXRα-LBD were exposed to the indicated peptide and compound for 12 h. Cell lysates were prepared and analysed by coIP. (**h**) Mutation of the LxxLL motif in BCR impairs its interaction with RXRα-LBD. BCR and BCR-LxxLL mutant were tagged with Flag epitope and transfected into A549 cells with Myc-RXRα-LBD. Cell lysates were prepared and analysed for interaction of BCR and its mutant with RXRα-LBD by coIP. (**i**) Trp305 is essential for RXRα-LBD binding to p85α-BCR. Myc-tagged RXRα-LBD or RXRα-LBD/W305Q was transfected together with HA-p85α-BCR into HEK293T cells in the presence or absence of 9-*cis*-RA (10^−7 ^M). Cell lysates were prepared and analysed by coIP. For western blotting, one of three or four similar experiments is shown.

tRXRα differs from RXRα in its ability to reside in the cytoplasm and interact with cytoplasmic p85α protein, an event that is inhibited by K-80003 (ref. 29). To address the role of tetramerization in modulating tRXRα interaction with p85α, we first determined how p85α binds to tRXRα. Thus, several p85α mutants (Fig. 4a) were constructed and analysed for their interaction with tRXRα. CoIP experiments showed that the N-terminal region of p85α, including the N-terminal SH3 and BCR domains, is sufficient for interacting with tRXRα, whereas the C-terminal region including NSH2, iSH2 and CSH2 domains was dispensable (Fig. 4b). We further observed that a p85α mutant encompassing only the BCR domain could interact with tRXRα and RXRα-LBD but not RXRα-A/B (Fig. 4c).

In an effort to understand the molecular basis for BCR interaction with tRXRα, we noticed the presence of two LxxLL motifs, ^161^LRQLL^165^ and ^240^LQYLL^244^, in BCR, which are commonly found in coactivators that mediates the transactivation of nuclear receptors^10^,^13^,^14^,^15^,^16^. Inspection of both motifs in the published BCR structure^36^ revealed that the ^240^LQYLL^244^ motif is buried in the central core of the BCR domain, while the ^161^LRQLL^165^ motif is located in a separate helix within a loop region. The ^161^LRQLL^165^ motif docks well to the coactivator-binding groove of RXRα (Fig. 4d), suggesting that the motif might be critical for RXRα binding. A peptide (BCR peptide) that encompasses the ^161^LRQLL^165^ motif (Fig. 4a) was therefore synthesized and examined for its binding to RXRα-LBD by Biacore assay. The peptide binds strongly to RXRα-LBD in the presence of 9-*cis*-RA with a *K*_d_ of 320 nM (Fig. 4e), which is in the range of coregulator peptide binding to nuclear receptor^37^. The role of the ^161^LRQLL^165^ motif was also illustrated by the enhancing effect of 9-*cis*-RA on p85α-BCR interaction with either RXRα-LBD or tRXRα (Fig. 4f). Furthermore, 9-*cis*-RA-induced RXRα-LBD interaction with p85α-BCR was inhibited by the LxxLL-containing BCR peptide conjugated with the cell-penetrating peptide derived from trans-activator of transcription (TAT), similar to the effect of K-80003, but not by the corresponding mutant peptide (Fig. 4g). Substitution of L164 and L165 in ^161^LRQLL^165^ motif with A also abolished the interaction of BCR with RXRα-LBD (Fig. 4h). Mutating W305 critical for the formation of the coactivator-binding groove^12^ to Q impaired the binding of RXRα-LBD with p85α-BCR either in the presence or absence of 9-*cis*-RA (Fig. 4i), even though the same mutation had no effect on RXRα-LBD interaction with RXRα-A/B (Fig. 3i). The interaction of the LxxLL-like motif in p85α with the coactivator-binding groove of tRXRα is biologically relevant as TNFα-induced activation of AKT in cells transfected with tRXRα and p85α was inhibited by cotransfection of BCR but not BCR mutant, similar to the inhibitory effect of K-80003 (Supplementary Fig. 7a). Exposure of cells to BCR peptide also resulted in a similar inhibition (Supplementary Fig. 7b). Altogether, these results demonstrate that the LxxLL motif in p85α can bind to the coactivator-binding groove of RXRα in analogy to the binding of transcriptional coactivators.

### Tetramerization prevents tRXRα from interacting with p85α

**Figure 5:**
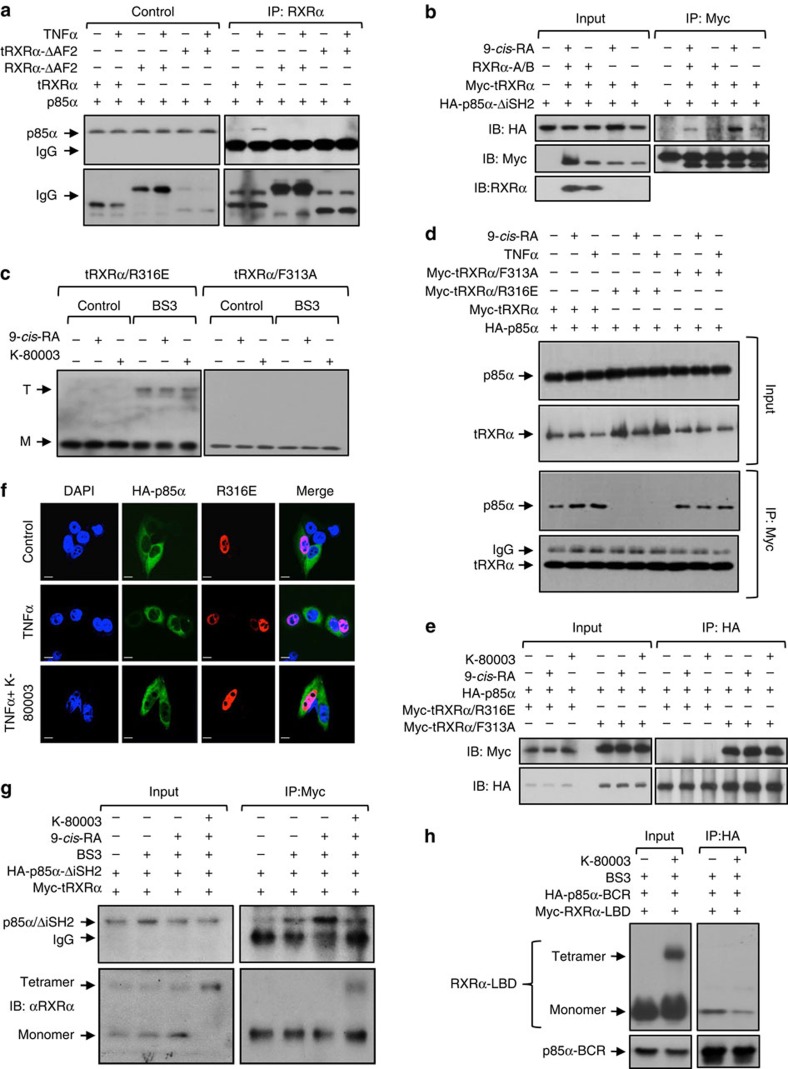
Tetramerization of tRXRα prevents its interaction with p85α. (**a**) AF2/H12 is required for binding p85α. HEK293T cells transfected with HA-p85α and Myc-tRXRα or mutants were treated with TNFα (10 ng ml^−1^) for 1 h, and analysed by coIP assay using anti-Myc antibody. (**b**) Regulation of tRXRα interaction with p85α by ligand and RXRα-A/B region. HEK293T cells transfected with the indicated HA-p85α-ΔiSH2, Myc-tRXRα and RXRα-A/B were treated with or without 9-*cis*-RA (10^−7 ^M) for 6 h, and analysed by coIP assay using anti-Myc antibody. (**c**) Tetramerization of tRXRα/R316E and tRXRα/F313A was analysed in MCF-7 cells transfected with tRXRα/R316E or tRXRα/F313A, which were then treated with 9-*cis*-RA (10^−7^ M) or K-80003 (5 × 10^−6^ M) for 6 h. Cell lysates were subjected to BS3 crosslinking and analysed by western blotting using ΔN197 anti-RXRα antibody. (**d**) Interaction of tRXRα/R316E and tRXRα/F313A with p85α. HEK293T cells transfected with the indicated expression plasmids were treated with TNFα (10 ng ml^−1^) or 9-*cis*-RA (10^−7 ^M) for 1 h, and analysed by coIP assay using anti-Myc antibody. (**e**) Characterization of p85α interaction with tRXRα/R316E or tRXRα/F313A. HEK293T cells transfected with the indicated expression plasmids were treated with 9-*cis*-RA (10^−7 ^M) or K-80003 (5 × 10^−6 ^M) for 6 h., and analysed by coIP assay using anti-HA antibody. (**f**) Mutation of R316 impairs tRXRα cytoplasmic localization. HEK293T cells cotransfected with Myc-tRXRα/R316E and HA-p85α were pretreated with K-80003 (5 × 10^−6 ^M) for 3 h before exposed to TNFα (10 ng ml^−1^) for 30 min. Cells were immunostained with anti-Myc and anti-p85α antibody, and visualized by confocal microscopy. Scale bar, 10 μm. (**g**) Tetramerization of tRXRα impairs its interaction with p85α. HepG2 cells transfected with Myc-tRXRα together with HA-p85α-ΔiSH2 were treated with K-80003 and or 9-*cis*-RA. Cell lysates were then subjected to BS3 crosslinking, and analysed by western blotting using anti-Myc antibody. (**h**) Tetramerization of RXRα-LBD impairs its interaction with p85α-BCR. HEK293T cells transfected with Myc-RXRα-LBD together with HA-p85α-BCR were treated with K-80003. Cell lysates were subjected to BS3 crosslinking, and analysed by western blotting using anti-HA antibody. For western blotting, one of three or four similar experiments is shown.

To further characterize the ‘coactivator-like’ binding of p85α, we studied the requirement of AF2/H12 in tRXRα, which is essential for the formation of the coactivator-binding groove^12^,^38^, for tRXRα interaction with p85α. As previously reported^29^, tRXRα interacted with the full-length p85α in the presence of TNFα. Removing AF2/H12 from tRXRα (tRXRα/ΔAF2) abolished its interaction with p85α (Fig. 5a). Interfering AF2/H12 activity by transfecting RXRα-A/B capable of binding AF2/H12 also inhibited 9-*cis*-RA-induced interaction of tRXRα with p85α-ΔiSH2 (Fig. 5b). These results further support the ‘coactivator-like’ binding of p85α.

Because the coactivator-binding groove of RXRα was shielded in the tetrameric form of RXRα-LBD (Fig. 1), our observation that the LxxLL-like motif in p85α interacted with the coactivator-binding groove of RXRα implied that the p85α-binding activity of tRXRα is impaired in its tetrameric form. To address this, we evaluated the binding of p85α by two tRXRα mutants, tRXRα/F313A and tRXRα/R316E, which exist in different oligomeric forms^25^. RXRα/F313A is a transcriptionally constitutively active mutant receptor with H12 adopting an active conformation to form the coactivator-binding groove^39^. The mutant does not exist as a tetramer as its H12 is not available to contribute to the formation of tetramers^25^. In contrast, tRXRα/R316E, a transcriptionally inactive mutant, exhibited predominantly as a tetramer^25^. In agreement with previous observations^25^, our cross-link experiments detected a strong tetrameric species of tRXRα/R316E but not tRXRα/F313A independent of the presence of 9-*cis*-RA or K-80003 (Fig. 5c). When both mutants were analysed for their interaction with p85α by coIP experiments, we found that tRXRα/R316E could not bind to p85α either in the absence or presence of TNFα or 9-*cis*-RA, whereas tRXRα/F313A showed a constitutive and 9-*cis*-RA-independent interaction with p85α compared to tRXRα (Fig. 5d). K-80003 also failed to modulate the interaction of both mutants with p85α (Fig. 5e). Immunostaining revealed lack of colocalization of the tRXRα/R316E mutant with p85α in the absence or presence of TNFα (Fig. 5f). To further determine the inhibitory effect of tRXRα tetramerization, we examined the interaction of p85α-ΔiSH2 with tRXRα tetramers crosslinked by BS3. Cells transfected with HA-p85α-ΔiSH2 and Myc-tRXRα were treated with 9-*cis*-RA and/or K-80003, and subsequently exposed to BS3. 9-*cis*-RA-induced tRXRα interaction with p85α-ΔiSH2 was potently inhibited by BS3 that stabilized tRXRα tetramers (Fig. 5g). Furthermore, the monomeric form but not the tetrameric form of RXRα-LBD interacted with p85α-BCR (Fig. 5h). These results together with our structural information demonstrate that K-80003 inhibits tRXRα interaction with p85α by promoting tRXRα tetramerization that masks the p85α-binding region on tRXRα.

### Tetramerization regulates tRXRα subcellular localization

**Figure 6:**
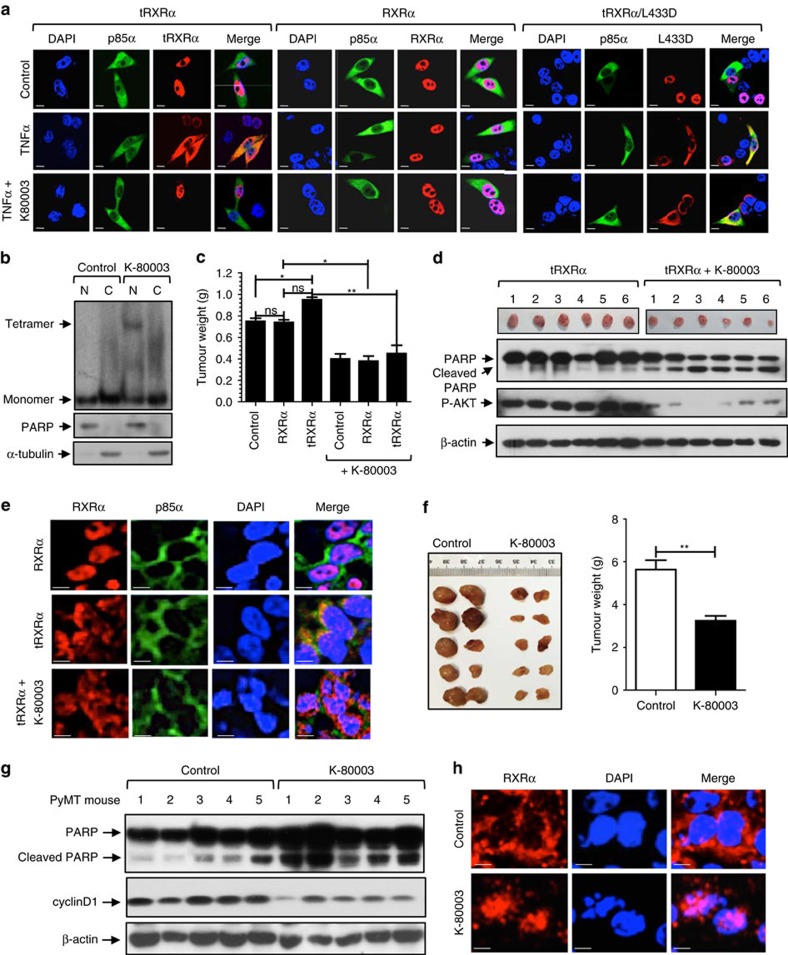
Effect of tetramerization on the subcellular localization of tRXRα. (**a**) Effect of TNFα and K-80003. MCF-7 cells cotransfected with Myc-RXRα, Myc-tRXRα, tRXRα/L433D and p85α were pretreated with or without K-80003 (5 × 10^−6 ^M) for 3 h before exposed to TNFα (10 ng ml^−1^) for 30 min. Cells were immunostained with anti-Myc and anti-p85α antibody, and their subcellular localization revealed by confocal microscopy. (**b**) Tetrameric tRXRα resides in the nucleus. HEK293T cells cotransfected with Myc-tRXRα were treated with or without K-80003 (5 × 10^−6 ^M) for 6 h. Nuclear (N) and cytoplasmic (C) fractions were prepared, subjected to BS3 crosslinking, and analysed by western blotting using anti-Myc antibody. The purity of fractions was examined by analysing the expression of nuclear PARP and cytoplasmic α-tubulin in non-crosslinked fractions. One of three similar experiments is shown. (**c**) Effect of K-80003 on the growth of MCF-7 cells in mice. Nude mice injected with MCF-7 cells stably transfected with control vector, Myc-RXRα, or Myc-tRXRα were administered with K-80003 (20 mg kg^−1^) for 12 days. *P<0.05; **P<0.01. (**d**) Effect of K-80003 on PARP cleavage and AKT activation in MCF-7 xenograft tumours. Lysates prepared from tumours from nude mice treated with vehicle or K-80003 were analysed by western blotting. (**e**) K-80003 alters the subcellular localization of tRXRα in MCF-7 xenograft tumour cells. Tumour sections prepared from nude mice treated with vehicle or K-80003 were immunostained with anti-Myc antibody. (**f**) Effect of K-80003 on the growth of MMTV-PyMT mammary tumour. Four-week old MMTV-PyMT mice were fed with or without diet containing K-80003 (100 mg kg^−1^) for 4 weeks, and the appearance of tumour was determined. **P<0.01. (**g**) Effect of K-80003 on PARP cleavage and cyclin D1 expression in MMTV-PyMT mammary tumour cells. Lysates prepared from tumours from MMTV-PyMT mice fed with vehicle or K-80003 were analysed by western blotting. (**h**) K-80003 induces RXRα nuclear localization in MMTV-PyMT tumour cells. Tumour sections from MMTV-PyMT mice fed with or without K-80003 (100 mg kg^−1^) for 4 weeks were immunostained with ΔN197 anti-RXRα antibody. Scale bar, 10 μm.

The interaction of tRXRα with p85α occurs in the cytoplasm^29^. We next determined whether K-80003-induced stabilization of tRXRα tetramers could modulate its subcellular localization. When transfected into cells, both tRXRα and RXRα resided mainly (>80%) in the nucleus. However, upon TNFα treatment tRXRα was found in the cytoplasm of cells (>65%), colocalizing extensively with p85α. In contrast, TNFα had no effect on the nuclear localization of RXRα. When cells were cotreated with K-80003, TNFα-induced colocalization of tRXRα with p85α in the cytoplasm was inhibited, resulting in tRXRα nuclear localization (Fig. 6a). The effect of K-80003 was likely due to its binding to tRXRα as the cytoplasmic colocalization of p85α with tRXRα/L433D mutant defective in K-80003 binding was not affected by K-80003. Thus, TNFα-induced cytoplasmic localization of tRXRα was likely due to its cytoplasmic retention by TNFα-activated p85α through protein/protein interaction, suggesting that tRXRα tetramers incapable of binding p85α might reside in the nucleus. To address this, extracts prepared from cells transfected with tRXRα were subjected to crosslinking by BS3. Nuclear and cytoplasmic fractions were then prepared and analysed. Western blotting showed that K-80003-stabilized tetrameric form of tRXRα was found exclusively in the nuclear fraction, while tRXRα monomer was distributed both in the nuclear and cytoplasmic fractions (Fig. 6b). Thus, K-80003-stabilized tRXRα tetramer is mainly nuclear, likely resulted from tRXRα dissociation from p85α or other cytoplasmic proteins.

To study whether the anti-tumour effect of K-80003 could be attributed to its effect on the subcellular localization of tRXRα *in vivo*, we stably transfected tRXRα or RXRα into MCF-7 cells, and the resulting stable clones (Supplementary Fig. 8a) were inoculated into nude mice. Overexpression of tRXRα but not RXRα in MCF-7 breast cancer cells enhanced AKT activation *in vitro* (Supplementary Fig. 8b) and promoted the growth of MCF-7 tumour in animals, which was suppressed when animal were treated with K-80003 (Fig. 6c; Supplementary Fig. 8c). Inhibition of the growth of MCF-7/tRXRα tumour by K-80003 was accompanied with reduced AKT activation and enhanced PARP cleavage (Fig. 6d). To determine whether the anti-cancer activity of K-80003 was associated with its modulation of tRXRα subcellular localization, tumour specimens from nude mice were analysed by RXRα immunostaining (Fig. 6e). While RXRα was nuclear, tRXRα was predominantly cytoplasmic. However, a significant amount of tRXRα was found in the nucleus when animals were administered with K-80003. We also used polyomavirus middle T antigen (PyMT) transgenic mice^40^ to study the anti-cancer effect of K-80003 and its modulation of the subcellular localization of tRXRα, which was highly expressed in PyMT mammary tumour developed in these mice (Supplementary Fig. 9a). K-80003 potently inhibited the growth of PyMT mammary tumour in this animal model (Fig. 6f), accompanied with induction of PARP cleavage and inhibition of cyclin D expression (Fig. 6g) as well as inhibition of tumour cell proliferation (Supplementary Fig. 9b). Immunostaining of PyMT tumour specimens using Δ197 anti-RXRα antibody that recognizes both RXRα and tRXRα revealed a predominant cytoplasmic RXRα staining (Fig. 6h). In contrast, RXRα staining was mainly found in the nucleus when mice were dosed with K-80003. Thus, K-80003 induction of tRXRα nuclear localization through its modulation of tRXRα tetramerization likely represents a major mechanism by which the compound exerts its potent therapeutic effect.

## Discussion

RXRα is unique in that it can form not only homodimers and heterodimers but also homotetramers^4^,^5^,^6^,^7^,^8^,^9^,^10^,^11^,^17^,^25^,^33^,^38^, suggesting that the equilibrium between these different oligomeric states plays a role in regulating RXRα functions. While the function and mechanism of RXRα homodimer and heterodimers are well documented, the role of RXRα homotetramers and its regulation remains poorly defined. We report here that K-80003 represents a unique RXRα ligand capable of shifting the oligomeric equilibrium by selectively binding to and stabilizing the tetrameric conformation. Our crystal structure of RXRα-LBD in complex with K-80003 shows that six molecules of K-80003 bind in a tetramer by a unique combination of distinct and canonical binding mechanisms acting like a ‘3-pronged’ binding mode. The comprehensive interactions between the bound K-80003 molecules and the protein provide a plausible explanation for how K-80003 binding can stabilize the tRXRα tetrameric state. Biochemical studies confirmed that K-80003 binding could promote tetramerization of tRXRα but not full-length RXRα. K-80003-induced stabilization of tRXRα tetramer was associated with inhibition of its interaction with p85α and activation of tRXRα-dependent PI3K/AKT signalling. The tetramerization also represents a crucial event in determining the subcellular localization of tRXRα. Collectively, these results unravel a critical role of tRXRα tetramerization in regulating its subcellular localization and nongenomic interaction with cytoplasmic signalling proteins.

Although how tRXRα binds to p85α remains to be precisely defined, our data demonstrated that an LxxLL motif in p85α and the amino acid residues at the coregulator-binding groove of RXRα are critical for their interaction (Fig. 4). These data in combination with information from comparing the RXRα-LBD dimer and RXRα-LBD/K-80003 tetramer crystal structures demonstrate that the potential p85α-binding site on tRXRα resides in the coregulator-binding groove. In the K-80003-stabilized RXRα-LBD tetramer, H12 protrudes into the coregulator-binding groove of the neighbouring monomer (Fig. 1), making the groove inaccessible for p85α binding (Supplementary Fig. 2). Modulation of gene transcription by nuclear receptors is mediated through their interaction with coactivators or corepressors via the LxxLL motif and the L/IxxI/VI motif, respectively^10^,^13^,^14^,^15^,^16^. Thus, our identification of a functional LxxLL motif in p85α suggests that p85α may serve as a nongenomic coregulator of tRXRα in analogy with the regulation of nuclear receptor transactivation by coregulators in the nucleus^10^,^13^,^14^,^15^,^16^. The genomic and nongenomic coregulators of RXRα may act coordinately to mediate the crosstalk between RXRα nuclear and cytoplasmic signallings. It is noteworthy that several nuclear receptors, including oestrogen receptor, glucocorticoid receptor, peroxisome proliferator-activated receptors, thyroid receptor and retinoic acid receptor could interact with p85α^41^,^42^,^43^,^44^,^45^,^46^,^47^,^48^,^49^. Whether p85α serves as a common coregulator for different nuclear receptors to modulate PI3K signalling remains to be seen. The concept of cytoplasmic nuclear receptor coregulators will expand our conventional view of coregulators and may lead to the identification of a class of cytoplasmic signalling proteins that mediate the nongenomic action of RXRα and perhaps other nuclear receptors.

Interaction between the N- and the C-terminal segments of a protein appears to be evolutionarily selected for some functional advantages^50^. Although the N-terminal A/B domains of nuclear receptors are intrinsically disordered, studies have now shown that they are capable of undergoing a disorder-to-order transition upon binding specific target molecules^3^. Thus far, the N/C intramolecular interaction has been described for several nuclear receptors, including oestrogen receptor^51^, progesterone receptor^52^, peroxisome proliferator-activated receptors^53^ and androgen receptor^34^. We report here that N/C interaction also occurs in RXRα and acts to mediate RXRα interaction with p85α, defining a regulatory paradigm for RXRα action. Although how the N/C interaction in RXRα is mediated remains unclear, our inspection of the sequences (^61^mgppfsvisspmgphsmsvp^80^) at the N-terminal A/B domain of RXRα (Fig. 3g) revealed the existence of potential polyproline II (PPII) domain characterized by a PxxP core motif known to bind to SH3, WW and EVH1 domain^54^. This is reminiscent of previous reports that proline-rich nuclear receptor co-regulatory protein 2 (PNRC2) uses a SH3 domain-binding motif (SEPPSPS) to interact with the LBDs of different nuclear receptors including oestrogen receptor, glucocorticoid receptor, progesterone receptor, thyroid receptor, retinoic acid receptor and RXR^55^, and that a unique interaction motif exists in the ligand-binding domain of oestrogen receptor for binding PPII-like motif^56^. Although AF2/H12 at the C-terminal end of RXRα is required for the N/C interaction (Fig. 3h), the coactivator-binding groove involving the AF2/H12 was not required (Fig. 3i). This is supported by the result that the N/C interaction was inhibited by 9-*cis*-RA (Fig. 3e). How the AF2/H12 engages the N/C interaction remains to be determined. The N-terminal A/B domain through intramolecular interaction with the C-terminal domain may allosterically affect the function of distant domains to ensure that RXRα activity is appropriately achieved. As the disruption of the N/C interaction has been implicated in the regulation of nuclear receptor activity, ligand sensitivity and subcellular localization^34^,^35^,^51^,^52^,^53^, our illustration of the existence of the N/C intramolecular interaction in RXRα provides a molecular explanation for the oncogenic effect of tRXRα in tumour cells^29^. It is noteworthy that in addition to various proteolytically cleaved RXRα products^57^,^58^,^59^,^60^,^61^,^62^,^63^,^64^, RXRα N-terminal splicing variants have been identified^65^,^66^. Furthermore, the N-terminal A/B domain is enriched with phosphorylation sites and several kinases have been identified to phosphorylate the region^67^,^68^. RXRα migrates from the nucleus to the cytoplasm in response to differentiation^69^, survival^29^,^70^, apoptosis^71^ and inflammation^29^,^70^,^72^. It remains to be determined if and how the N/C interaction in RXRα plays a role in mediating the crosstalk between RXRα and signal transduction pathways under both physiological and pathophysiological conditions.

In conclusion, our results elucidate a previously unrecognized role of RXRα tetramers and demonstrate that conformational selection plays a critical role in the regulation of the nongenomic function of RXRα. We showed that the tetramerization of RXRα could be regulated by several mechanisms including ligand binding, intra domain–domain interaction and nongenomic interaction with cytoplasmic signalling proteins. This study opens an opportunity to develop novel RXRα-based therapeutics by selectively stabilizing a particular oligomeric state. The selective induction by K-80003 of the tetramerization of tRXRα but not RXRα suggests that this class of compounds may preferentially affect tRXRα activity, which is therapeutically desirable since tRXRα is often overproduced in cancer cells^29^.

## Methods

### Protein expression and purification

The human RXRα LBD (residues Thr223 to Thr462) cloned as an N-terminal histidine-tagged fusion protein in pET15b expression vector was expressed in *Escherichia coli* BL21 strain (Stratagene). After sonication, cell extract was incubated with the His60 Ni Superflow resin, and His-tagged RXRα-LBD-resin complexes were washed, eluted and concentrated to 5 mg ml^−1^ for subsequent trails^73^,^74^. For crystallization experiment, bovine thrombin (Sigma) was used to cleave the His tag from the purified His-tagged RXRα-LBD. The resulting His tag was removed on the Resource-Q column (GE) using 0.1–1 M NaCl gradient and the Tris-Cl pH 8 buffer. RXRα-LDB was further purified using gel filtration on a Superdex 200 2660 column (GE) pre-equilibrated with the 75 mM NaCl, 20 mM Tris-Cl buffer (pH 8).

### Crystallization and structure solution

Crystallization conditions were initially determined using the sitting-drop vapor-diffusion method and the crystallization screens Index and PEG-Ion solutions from Hampton Research. Final crystals were obtained by cocrystallizing the protein and the ligand. The protein-ligand complex contained 0.35 mM of RXRα-LBD, 0.5–0.7 mM of a ligand, 1% DMSO, 100 mM NaCl and 20 mM Tris-Cl buffer (pH 7.6). 0.2 μl of the protein-ligand complex was mixed with 0.2 μl of the well solution (20% PEG3330 and 0.2 M Na Acetate) and incubated at 20 °C. Crystals appeared in 5–10 days and grew into 0.2 × 0.1 × 0.1 mm prisms. The crystals were flash-cooled in mother liquor containing 1.5 mM of K-80003 and 20% glycerol as a cryoprotectant. Diffraction data was collected from the cryo-cooled crystal (100 K) on an in-house diffractometer (Rigaku X-ray generator with a rotating anode, VariMax optic and R-AXIS HTC detector). Data was processed using the programme iMOSFLM, part of the CCP4 suite^75^. The b-factor of the data set according to the Wilson plot was 60 Å^2^.

The crystal structures were determined using the molecular replacement program Phaser^76^ with pdb entry 4N8R as an initial model. Coot^77^ and the program suite Phenix^78^ were used for the model rebuilding and refinement. Parameter files and initial models for the ligands were prepared by eLBOW of Phenix. The data collection and refinement statistics are summarized in Table 1. Overall, 96.5% of residues in the refined structure are in favored regions of the Ramachandran plot, and no residues fall into disallowed regions. Coordinate errors, as estimated by Phenix, is 0.37 (Å).

### Plasmid constructions

Expression plasmids Myc-RXRα, Myc-RXRα-Δ40, Myc-RXRα-Δ60, Myc-tRXRα, Myc-RXRα-Δ100, Myc-RXRα-ΔAF2, Myc-RXRα-ΔA/B, Myc-RXRα-ΔA/BΔAF2, Myc-RXRα-LBD, Myc-tRXRα-ΔAF2, Myc-RXRα-1-235, Myc-RXRα-A/B, Myc-RXRα-LBD/W305Q, Myc-tRXRα/R316E, Myc-tRXRα/F313A, HA-RXRα-A/B, HA-RARγ, HA-p85α, HA-p85α-ΔiSH2, HA-p85α-NIC, HA-p85α-SH3, HA-p85α-BCR, Flag-p85α-BCR, Flag-p85α-BCR LxxAA mutant and bacteria expressed His-RXRα-LBD, His-RXRα-LBD/L433D, His-RXRα-LBD/R316E, His-RXRα-LBD/Q275E, His-RXRα-LBD/F439A were constructed with standard methods (see Supplementary Table 1 for primer sequences).

### Peptide synthesis

Peptides were synthesized on MBHA resin using Fmoc synthesis and DIC/HOBt coupling with an Advanced Chem Tech 350 and 396 multiple peptide synthesizer^79^. Cell-penetrating peptide derived from TAT (GRKKRRQRRRPPQ) was conjugated with p85α-peptide^79^. All peptides were acetylated on their N termini, and all were amidated on their C termini. Standard deprotection conditions were used for all peptides. Peptides were purified by HPLC on C18 columns and confirmed by MALDI mass analysis.

### Gel filtration

Gel filtration was carried out using an AKTA FPLC system with HiLoad 16/600 Superdex 200 pg (GE Healthcare Life) pre-equilibrated with Binding buffer (50 mM Tris-Cl (pH 7.4), 200 mM NaCl) at a flow rate of 1 ml min^−1^. Purified RXRα-LBD incubated with K-80003 or 9-*cis*-RA for 2 h was then subject to gel filtration chromatogram assay. Elutes of RXRα-LBD protein were separated by 8% non-denaturing PAGE followed by silver staining.

### Chemical crosslinking

Cells were transfected with RXRα or mutant expression vectors for 24 h and then exposed to 9-*cis*-RA (10^−7^ M) or K-80003 (5 × 10^−6^ M) for 6 h. Cell lysates were then prepared and incubated with bis[sulfosuccinimidyl] suberate (BS3, Pierce) dissolved in dimethyl sulfoxide to a final concentration of 1 mM. The reactions were stopped with protein gel loading buffer. Samples were resolved by SDS/PAGE in 8% acrylamide gels and protein bands were visualized by western blotting.

### Non-denaturing gel electrophoresis

Purified RXRα-LBD protein (0.2 μg μl^−1^) was incubated with DMSO, 9-*cis*-RA (0.5 μM), and/or K-80003 (20 μM) for 3 h at 4 °C in a total volume of 20 μl, and proteins were separated by 8% non-denaturing PAGE followed by Coomassie Bright Blue staining.

### Cell culture and transfection

MCF-7 breast cancer cells, HepG2 liver cancer cells, A549 lung cancer cells, and HEK293T human embryonic kidney cells (from ATCC) were cultured in Dulbecco’s modified Eagle’s medium containing 10% fetal bovine serum. The cells were maintained at 5% CO_2_ at 37 °C. Subconfluent cells with exponential growth were used throughout the experiments. Cell transfections were carried out by using Lipofectamine 2000 (Invitrogen) according to the instructions of the manufacturer.

### Compound binding

Binding of compounds to RXRα-LBD or mutant was studied in phosphate buffer at 25 °C using isothermal titration calorimetry (ITC) assay that measures the heat either released or absorbed during binding. The purified His-tagged RXRα-LBD or mutant protein (50 μM in 25 mM Tris-HCl, pH 7.5, 150 mM NaCl, 0.1% DMSO) was placed in the sample cell of MicroCal VP-ITC titration calorimeter. The compounds to be studied were diluted to a concentration of 1 mM in the same buffer. All solutions were degassed before the titrations. Titration was carried out using a 2-μl compound with injection time 4 s and a 120 s delay between each injection. The heat of dilution was obtained by injecting compounds into the same buffer and subtracted from the reaction before the fitting process. Calorimetric data were analysed using MicroCal Origin software (version 7.0).

### Peptide binding

Binding of BCR peptide to His-tagged RXRα-LBD was analysed at 25 °C by surface plasmon resonance (SPR) using a BIAcore T200 machine with CM5 chips (GE Healthcare). The purified His-tagged RXRα-LBD protein (20 μg ml^−1^ in 10 mM sodium acetate, pH 5) in the presence of 9-*cis*-RA was immobilized on the CM5 chip via amine coupling of RXRα-LBD’s –NH_2_ groups according to the manufacturer’s instructions. BCR peptide with different concentrations was injected into the chip. The sensor surface was regenerated with Glycine–HCl (10 mM, pH 2.5) when the data collection was finished in each cycle. Sensorgrams were fit globally with BIAcore T200 analysis using 1:1 Langmuir binding mode. The equilibrium dissociation constant (*K*_d_) was determined using BIAcore’s evaluation software provided by the manufacturer.

### Western blotting

Proteins or cell lysates were electrophoresed by SDS-PAGE gel and transferred to polyvinylidene difluoride (PVDF) membrane. The membranes were blocked with 5% skimmed milk in TBST (50 mM Tris-HCl (pH 7.4), 150 mM NaCl and 0.1% Tween20) for 1 h, then incubated with primary antibodies and secondary antibodies and detected using ECL system (Thermo). The dilutions of the primary antibodies were anti-RXRα (ΔN197, Santa Cruz) in 1:1,000, anti-PARP (H-250, Santa Cruz) in 1:3,000, anti-p85α (Millipore) in 1:1,000, anti-p-AKT (D9E, Cell Signaling Technology) in 1:1000, anti-AKT1/2/3 (H-136, Santa Cruz) in 1:1,000, anti-β-actin (Sigma) in 1:5,000, anti-c-myc (9E10, Santa Cruz) in 1:3,000, anti-HA (F-7, Santa Cruz) in 1:3,000, anti-Flag (F1804, Sigma) in 1:3,000. Images of all uncropped western blots can be found in Supplementary Figs 10–14.

### Coimmunoprecipitation assay

For coIP assay^29^, HEK293T cells grown in 10 cm dishes were transfected with various plasmids. Cells were lysed in 1 ml of lysis buffer (20 mM Tris (pH 7.5), 150 mM NaCl, 1% Triton X-100, 1 mM EDTA, 30 mM NaF, 2 mM sodium pyrophosphate) with a cocktail of proteinase inhibitors (Roche). Lysates were incubated with the appropriate antibody for 12 h at 4 °C and subsequently incubated with protein A-Sepharose beads for 1 h. The immunoprecipitates were collected and washed three times with lysis buffer. The protein–antibody complexes recovered on beads were subjected to immunoblotting using appropriate antibodies after separation by SDS-PAGE. Input represents 5% of cell lysates used for coIP assays.

### Confocal microscopy

Cells mounted on glass slides or tumour tissue frozen sections (5-μm-thick) were washed with PBS and fixed in 4% paraformaldehyde (PFA) for 15 min and permeabilized with PBS containing 0.1% Triton X-100 for 15 min. Fixed cells or tumour tissues were blocked with 5 mg ml^−1^ BSA in PBS for 30 min at room temperature, followed with incubation with primary antibodies for 3 h and secondary antibodies at room temperature for 1 h and co-stained with 4′6′-diamidino-2-phenylindole (DAPI) to visualize nuclei. The images were taken under an LSM-510 confocal laser scanning microscope system (Carl Zeiss).

### Animal studies

For nude mice xenograft study, Myc-RXRα and Myc-tRXRα cloned into pcdna3.0 vector were stably transfected into MCF-7 cells, and the resulting stable clones and control clone (2 × 10^6^ cells in 100 μl) were injected subcutaneously into nude mice (BALB/c, SPF grade, 16–18 g, 4–5-week old). For drug treatment, mice were intraperitoneally injected (i.p.) daily with K-80003 (20 mg kg^−1^) diluted in Tween-80 or vehicle (tween-80). Body weight and tumour size were measured every 2 days. Mice were killed after 12-day drug treatment and the tumours removed for various assessments. For MMTV-PyMT transgenic mouse study, 4-week old female mice of a transgenic mouse on the FVB/N genetic background expressing the PyMT oncogene under the control of MMTV LTR promoter^40^ were fed with diet containing with or without K-80003 (100 mg kg^−1^) for 4 weeks. Estimation of tumour appearance was performed by palpation once every day. Tumour tissues of left thoracic mammary glands were excised after the treatment. Portion of tissues was used to prepare extracts for western blotting, while the rest was fixed in 4% phosphate-buffered paraformaldehyde, and used for immunohistochemical staining using anti-Ki67 antibody and immunostaining using Δ197 anti-RXRα antibody. All experimentations and animal usage were performed and approved by the Animal Care and Use Committee of Xiamen University.

### Data analyses

Data were expressed as means±s.d. from three or more experiments. Statistical analysis was performed using Student’s *t*-test. Differences were considered statistically significant with *P*<0.05.

### Data availability

Coordinates and structure factors for the complex of RXRα-LBD/K-80003 have been deposited in the Protein Data Bank under accession code PDB 5TBP. The rest of the data that support the conclusions of this study are available from the corresponding authors upon reasonable request.

## Additional information

**How to cite this article:** Chen, L. *et al*. Modulation of nongenomic activation of PI3K signalling by tetramerization of N-terminally-cleaved RXRα. *Nat. Commun.* **8,** 16066 doi: 10.1038/ncomms16066 (2017).

**Publisher’s note:** Springer Nature remains neutral with regard to jurisdictional claims in published maps and institutional affiliations.

## Supplementary Material

Supplementary Information

## Figures and Tables

**Table 1:** Data collection and refinement statistics.

	**RXRα-LBD/K-80003**
*Data collection*	
Space group	*P*2_1_
Cell dimensions	
*a*, *b*, *c* (Å), *β* (°)	46.6, 99.4, 109.9, 99.2
Resolution (Å)	34–2.6 (2.7–2.6)*
*R*_merge_	0.080 (0.96)
*I*/σ*I*	6.1 (1.2)
Completeness (%)	100 (100)
Redundancy	3.7 (3.5)
	
*Refinement*	
Resolution range (Å)	34–2.6
No. reflections work set (*R*_FREE_ set)	30,186 (1,341)
*R*_WORK_/*R*_FREE_	0.195/0.242
No. of atoms	
Protein	6,618
Ligands	194
Water	134
*B*-factors (Å^2^)	
Protein	66.4
Ligands	85
Water	53
R.m.s. deviations	
Bond lengths (Å)	0.002
Bond angles (°)	0.61

^*^Highest-resolution shell is shown in parentheses.
