# Supplementary material for: Modulation of nongenomic activation of PI3K signalling by tetramerization of N-terminally-cleaved RXRα
Source: Nat Commun. 2017 Jul 17;8:16066. doi: 10.1038/ncomms16066 (PMC5520057; doi:10.1038/ncomms16066)
Supplement: Supplementary Information [file ncomms16066-s1.pdf]

Type of file: PDF
Size of file: 0 KB
Title of file for HTML: Supplementary Information
Description: Supplementary figures and supplementary table.

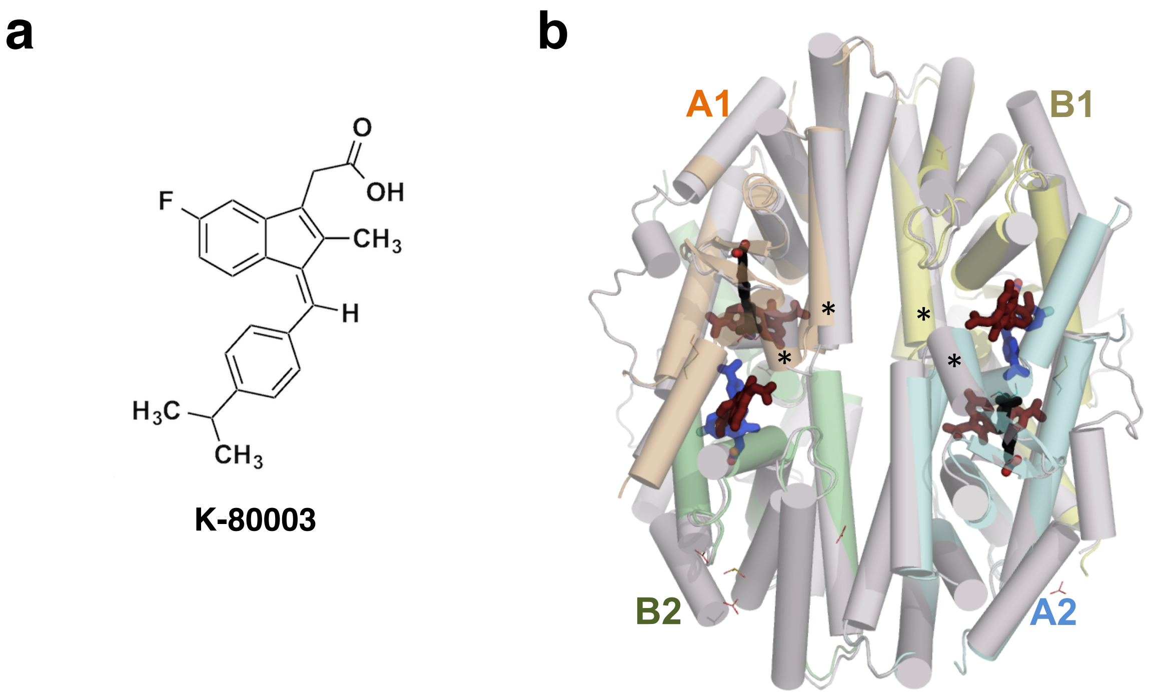

Supplementary Figure 1. Structure of RXRα-LBD in complex with K-80003
(a) Chemical structure of K-80003.
(b) Superposition of K-80003 bound RXRα-LBD tetramer (same colors as in Fig. 1a) and RXRα-LBD/tRA-isomer (grey cartoon/black sticks, PDB entry 1G5Y) RMS deviation for Cα atoms between PDB entries 1G5Y and 1G1U is 0.20 A. RMS deviation for Cα atoms between the K-80003-complex and 1G1U (or 1G5Y) is 1.20 A. The noteablet changes (marked with *) are around the tetramer interface.

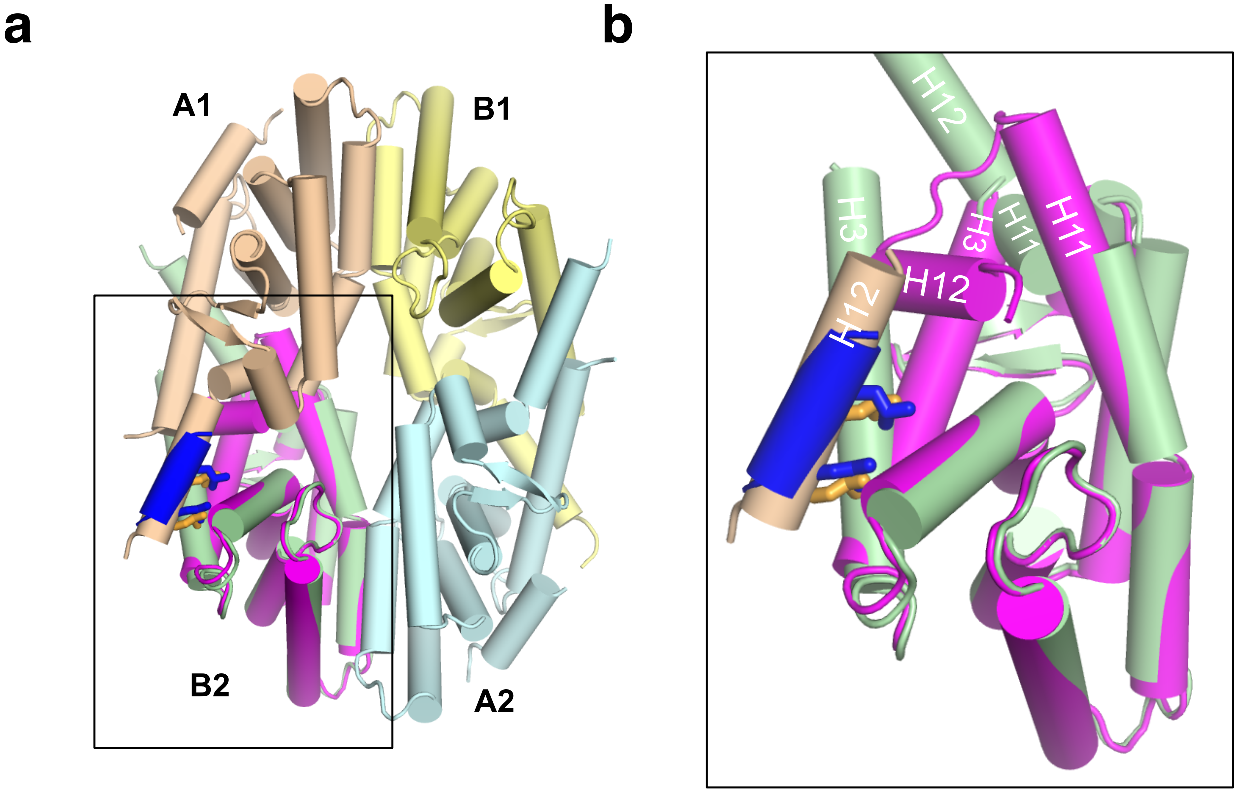

Supplementary Figure 2. Superposition of the agonist and coactivator peptide bound RXRα-LBD.

(a) Superposition of the agonist and coactivator peptide bound RXRα-LBD (PDB entry 3FUG) shown as magenta cartoon with one subunit of K-80003 bound RXRα-LBD tetramer (same orientation and same colors as in the Fig. 1a).

(b) Enlarged area around the superimposed molecules. The bound coactivator peptide in 3FUG is shown in blue, occupying a region that overlaps with H12 from the adjacent monomer. Hydrophobic side chains of the LMEML portion of the H12 (shown in orange) and the LxxLL motif of the coactivator peptide (shown in blue) are shown in sticks.

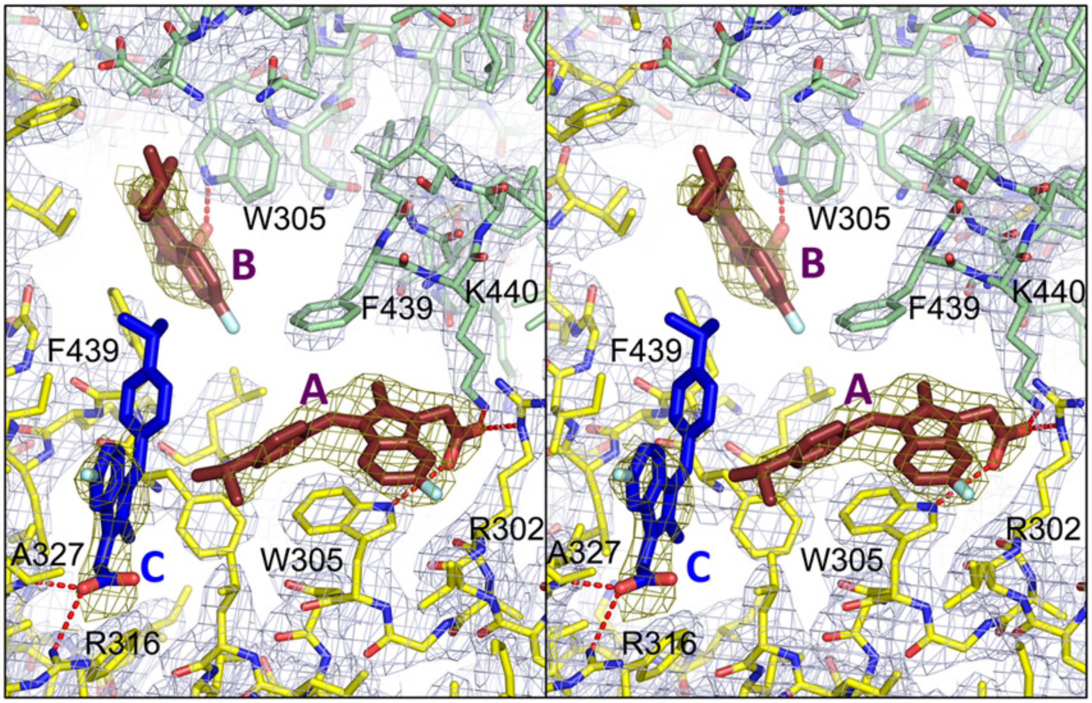

Supplementary Figure 3. Stereo view of the electron density map (2Fo-Fc, 0.8σ cut-off) around three K-80003 molecules that bind to the cavity in subunit A1/B2 (A1, green; a B2, yellow). The density mesh for the protein and the ligands have different colors for clarity. K-80003A and K-80003B (brown sticks) are bound to symmetry related sites at the interface between A1 and B2. Hydrogen bonds from the K-80003 molecules to protein are shown with red dash lines. K-80003C (blue) is bound to the site similar to the canonical ligand-binding site.

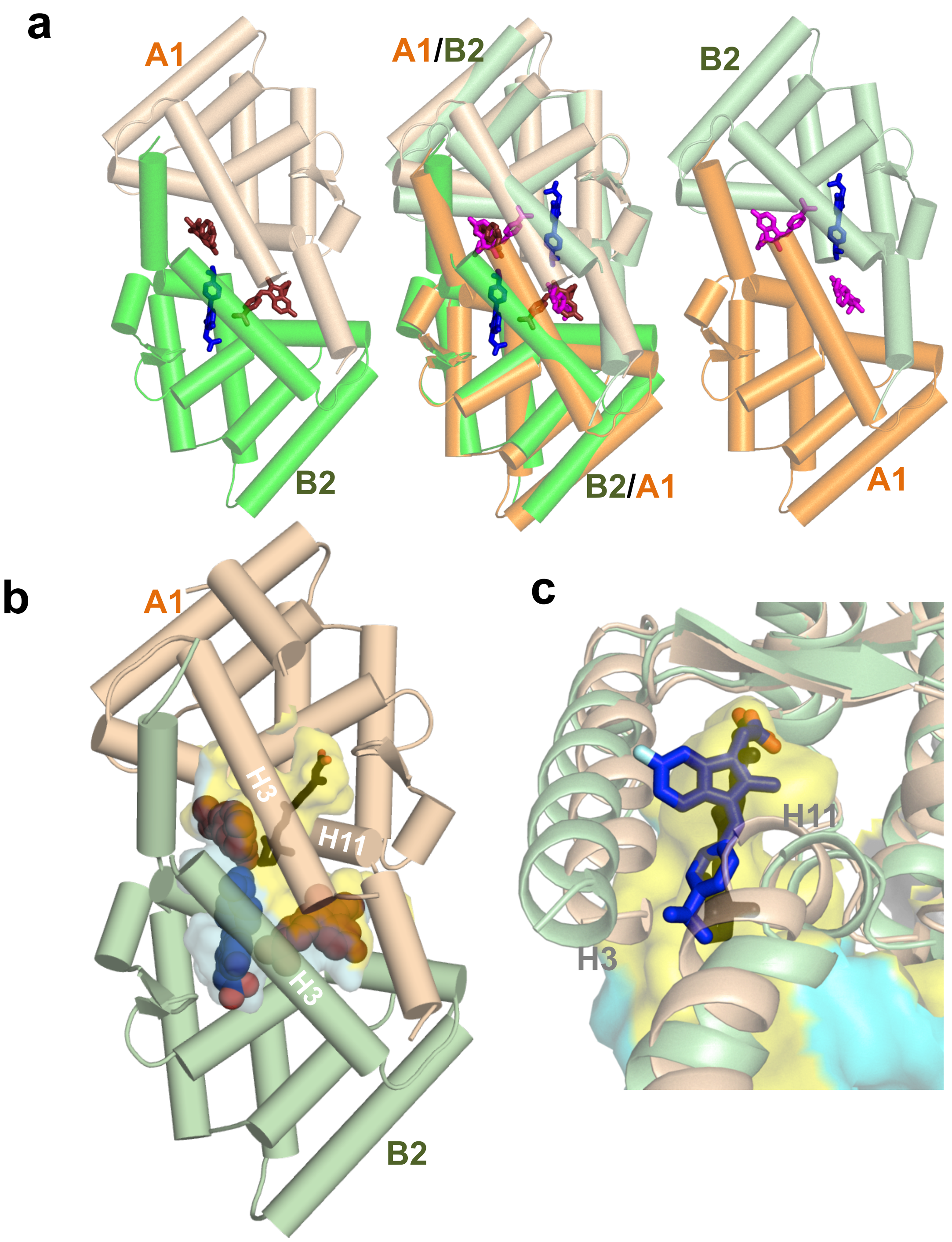

Supplementary Figure 4. Asymmetry of the cavity in A1/B2 subunit of the RXRα-LBD tetramer.
(a) Superposition of A1 andB2 subunits of the RXRα-LBD tetramer on themselves. K-80003 is shown as sticks. Colors are the same as Fig. 1b.
(b) Superposition of the molecule tRA-isomer (black sticks) from RXRα-LBD tetramer (pdb entry 1G5Y) on the A1/B2 subunit with bound K-80003 (spheres). The ligand-binding cavity is shown as a VDW surface.
(c) The superposition of the subunit B2 on A1 enlarged around the binding site for tRA-isomer (black sticks). The helices H3 and H11 of the subunit A1 (wheat) are much closer to each other than in the subunit B2 (green). As a result, the cavity of the subunit A1 is capable to bind tRA-isomer (black sticks) but does not fit K-80003 (blue sticks).

a

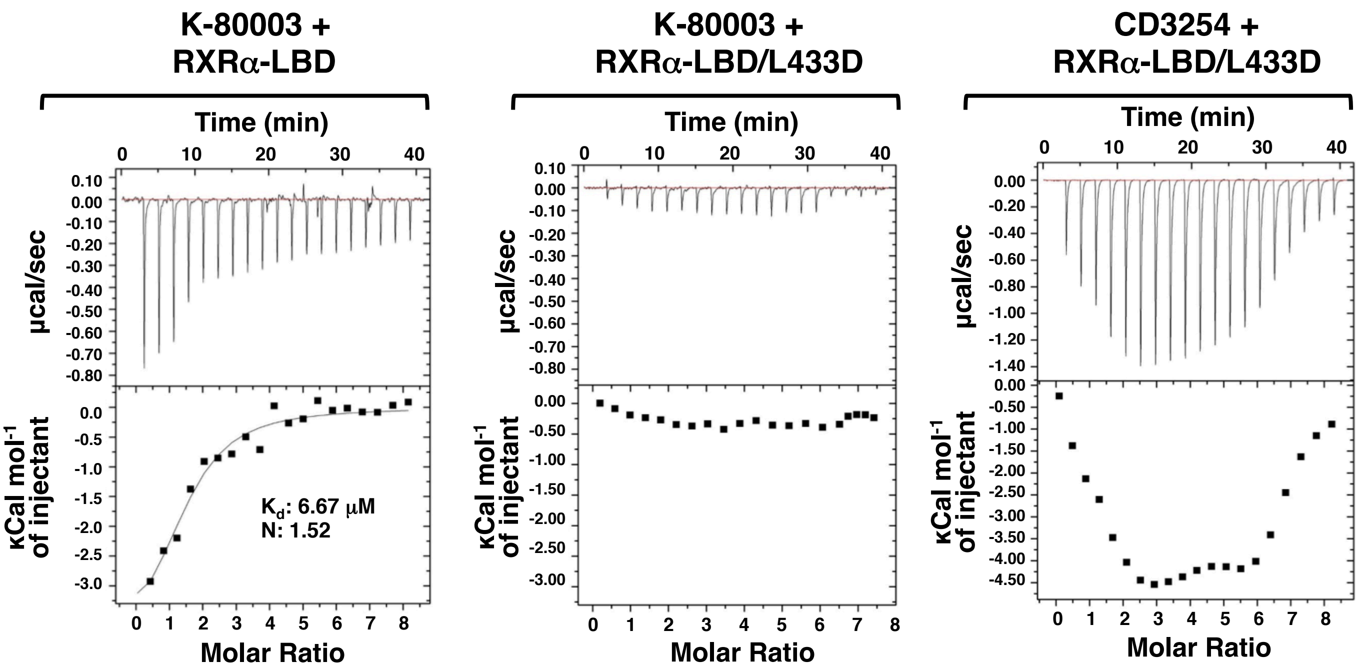

b

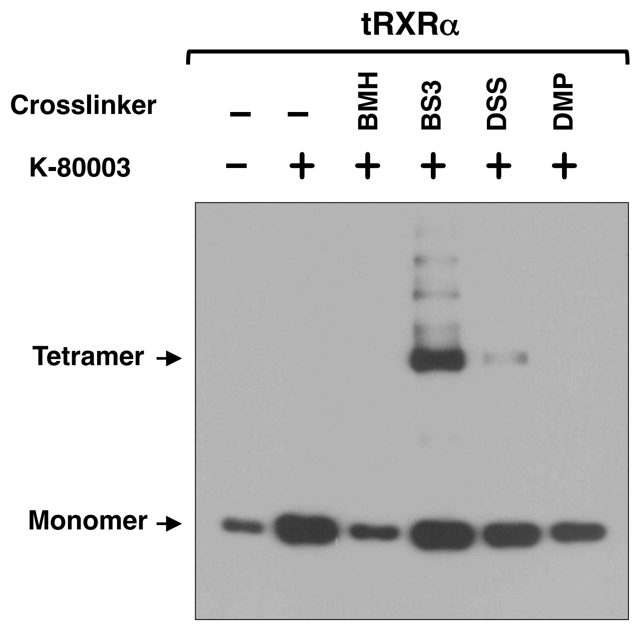

Supplementary Figure 5. Characterization of RXRα mutant and chemical crosslinkers.

(a) Analysis of RXRα-LBD/L433D for binding to K-80003 and RXRα agonist CD3254 by ITC assay. Data acquisition and analysis were performed using MicroCal Origin software (version 7.0).

(b) Characterization of chemical crosslinkers for their effect on tRXRα oligomerization. HEK293T cells transfected with Myc-tRXRα were treated with or without K-80003. Cell lysates were subjected to crosslink by the indicated chemical crosslinkers as recommended by the manufacturer, and analyzed by Western Blotting using anti-Myc antibody. HMH:bismaleimidohexane; BS3: bis(sulfosuccinimidyl)suberate; DSS: disuccinimidyl suberate; DMP: dimethyl pimelimidate,

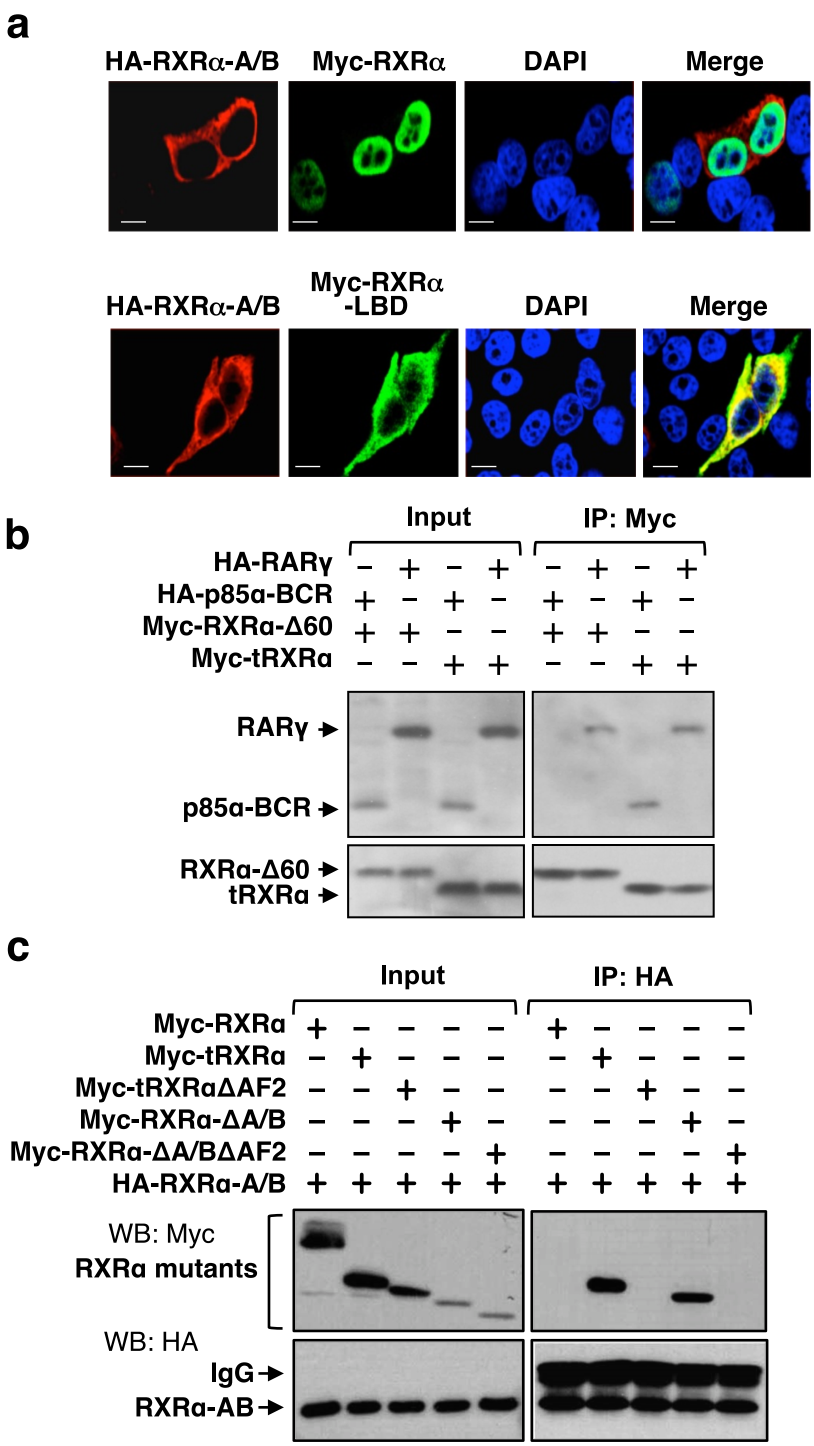

Supplementary Figure 6. Characterization of RXRα A/B domain and the LxxLL motif in p85α.

(a) Colocalization of RXRα A/B domain with RXRα-LBD but not RXRα revealed by immunostaining. HEK293T cells cotransfected with the indicated expression vectors were immunostained with anti-Myc or anti-HA antibody, and examined by confocal microscopy. Scale bar, 10 μm.

(b) RXRα-Δ60 interacts with RARγ but not RXRα-A/B. RXRα-Δ60 and tRXRα tagged with Myc epitope were transfected into HEK293T cells with HA-p85α-BCR or HA-RARγ, and their interaction was analyzed by coIP using anti-Myc antibody.

(c) AF2 in RXRα is required for binding A/B region. HA-RXRα-A/B and Myc-tagged RXRα mutants were transfected together in to HEK293T cells, and their interaction was analyzed by coIP.

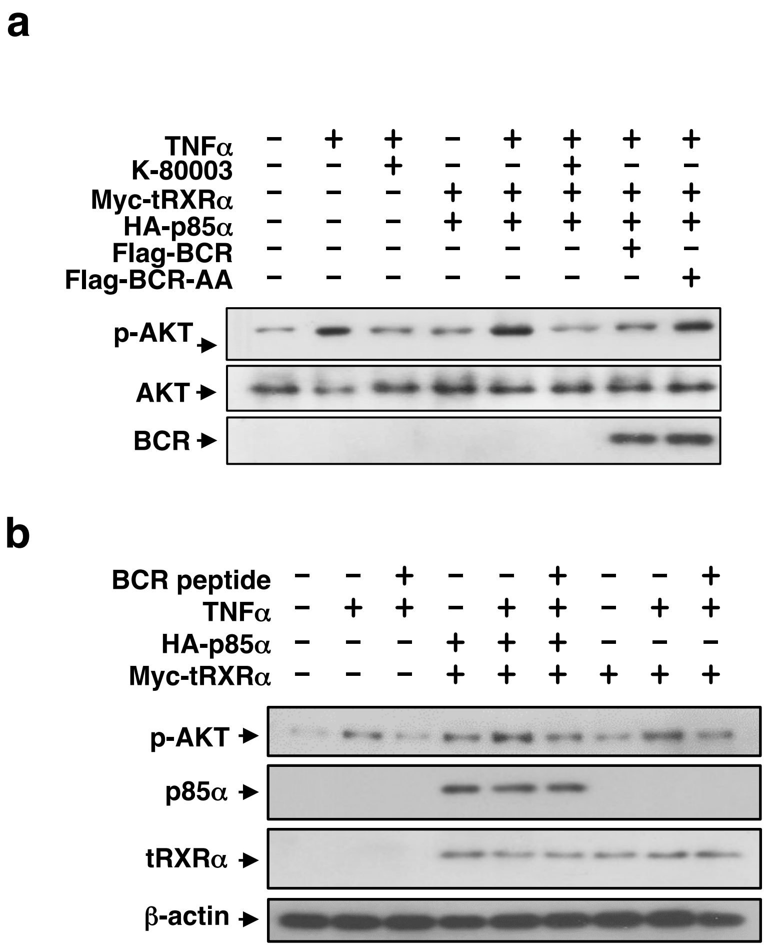

Supplementary Figure 7. Characterization of the LxxLL motif in BCR of p85α.

(a) Dominant-negative effect of BCR on tRXRα activation of AKT. A549 cells were transfected with the indicated expression vector prior to exposure to K-80003 ($5X10^{-6}$ M) for 3 h. Phosphorylated AKT and total AKT were analyzed by Western blotting.

(b) BCR peptide inhibits AKT activation. A549 cells were transfected with HA-p85α and/or tRXRα prior to exposure to BCR peptide for 12 h, and analyzed by Western blotting.

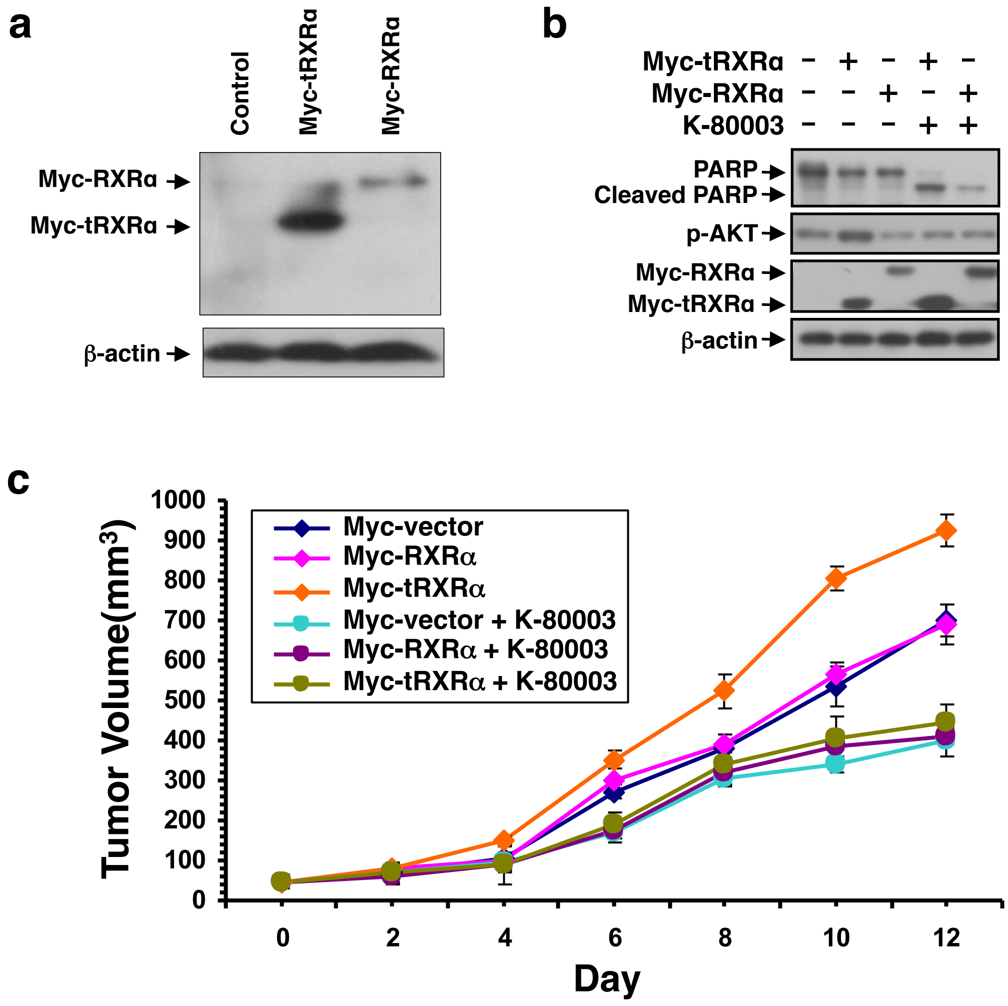

Supplementary Figure 8. Characterization of the inhibitory effect of K-80003 on the tumor promoting activity of tRXRα in nude mice.

(a) MCF-7 cells stably expressing tRXRα and RXRα analyzed by Western blotting.

(b) Effect of K-80003 on PARP cleavage and AKT activation in MCF-7 cells expressing tRXRα or RXRα. Cells stably expressing either RXRα or tRXRα were treated with or without K-80003 for 24 h, and analyzed by Western blotting.

(c) Effect of K-80003 on the growth of MCF-7 cells in nude mice. MCF-7 cells stably expressing tRXRα or RXRα were injected into nude mice. Mice were then administered with K-80003 (20 mg/kg), and the growth of tumor measured. Data are presented as the mean ± standard deviation (n=6 in each group).

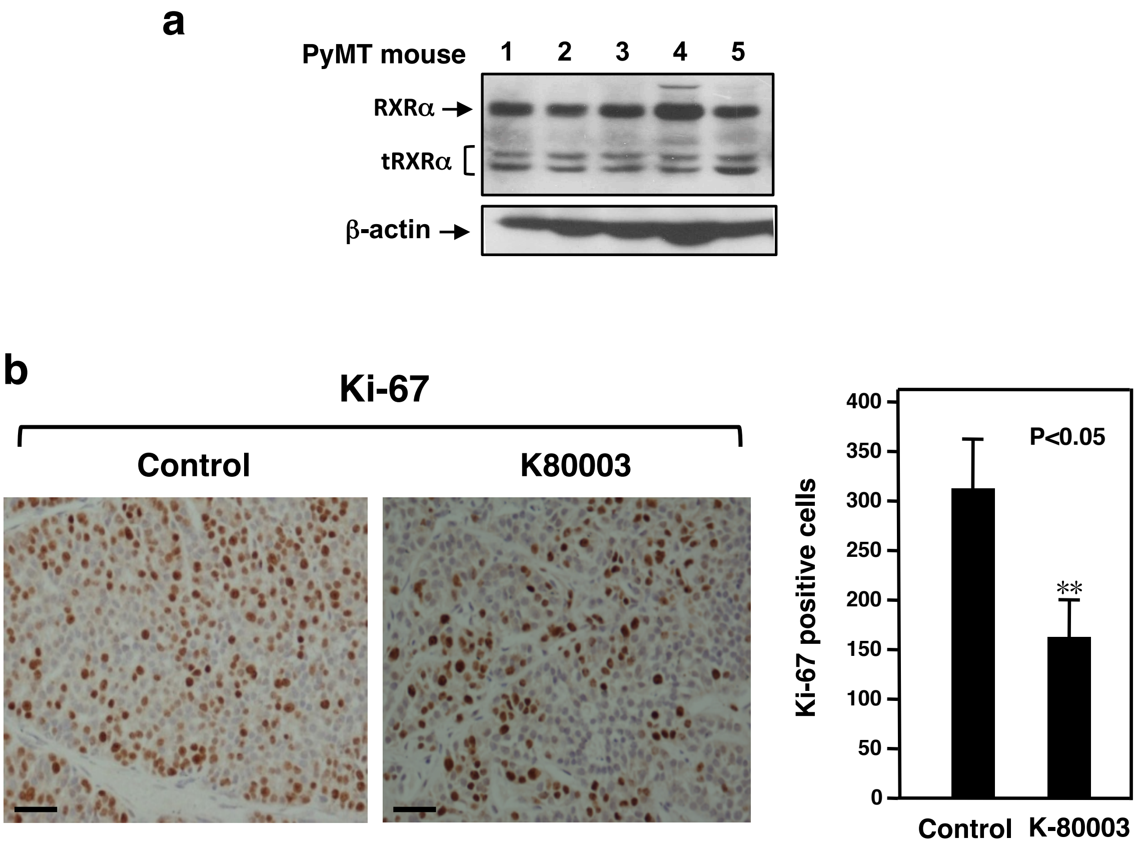

Supplementary Figure 9. Characterization of the inhibitory effect of K-80003 on the growth of mammary tumor in MMTV-PyMT transgenic mice.

(a) Expression of tRXRα in MMTV-PyMT mammary tumors analyzed by Western blotting using ΔN197 anti-RXRα antibody.

(b) Tumor sections from MMTV-PyMT mice fed with vehicle or K-80003 were immunostained with anti-Ki-67 antibody. Ki-67 positive cells were scored from 500 cells. Scale bar, 20 μm.

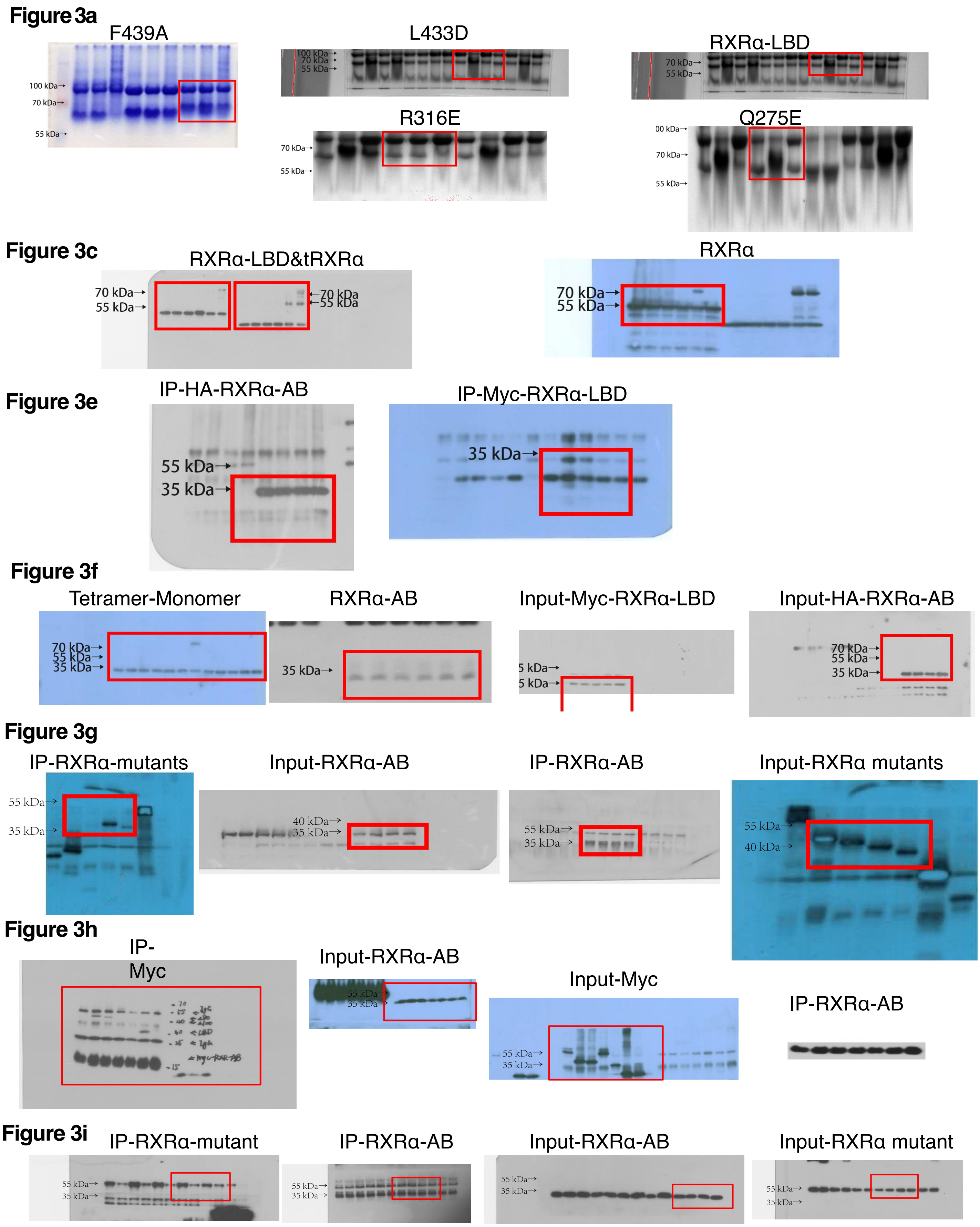

Supplementary Figure 10. Uncropped Western blot images for Figure 3.

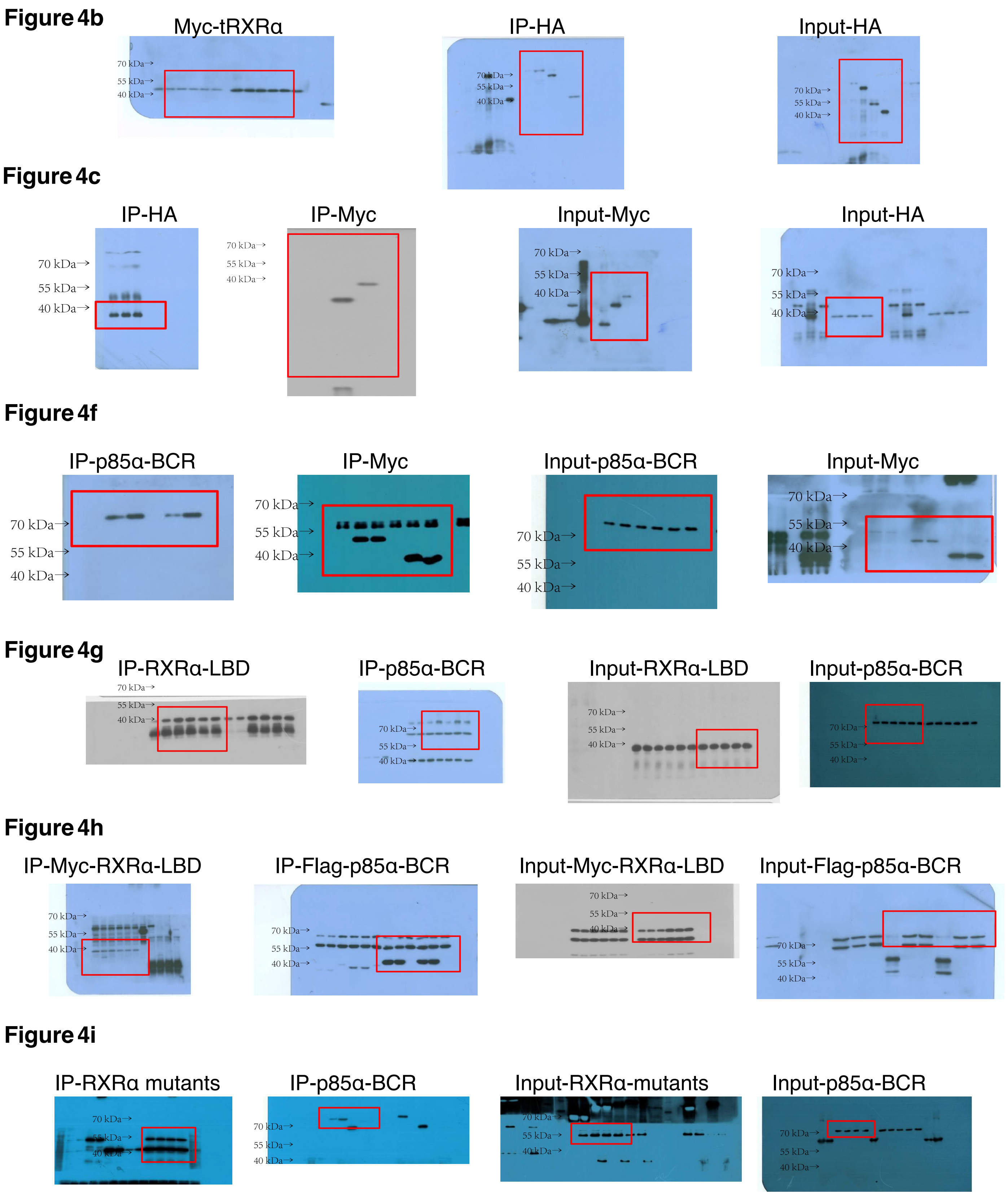

Supplementary Figure 11. Uncropped Western blot images for Figure 4.

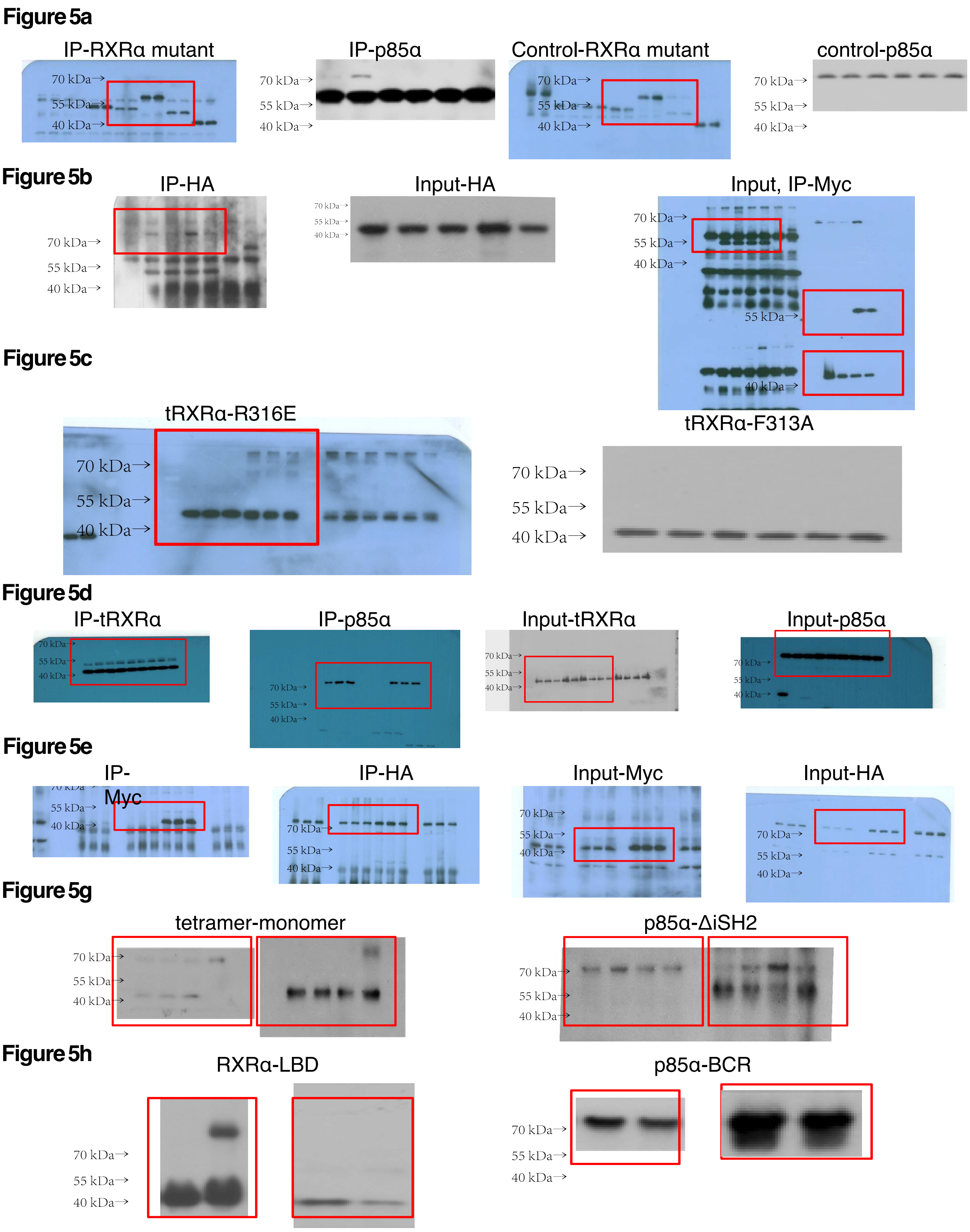

Supplementary Figure 12. Uncropped Western blot images for Figure 5.

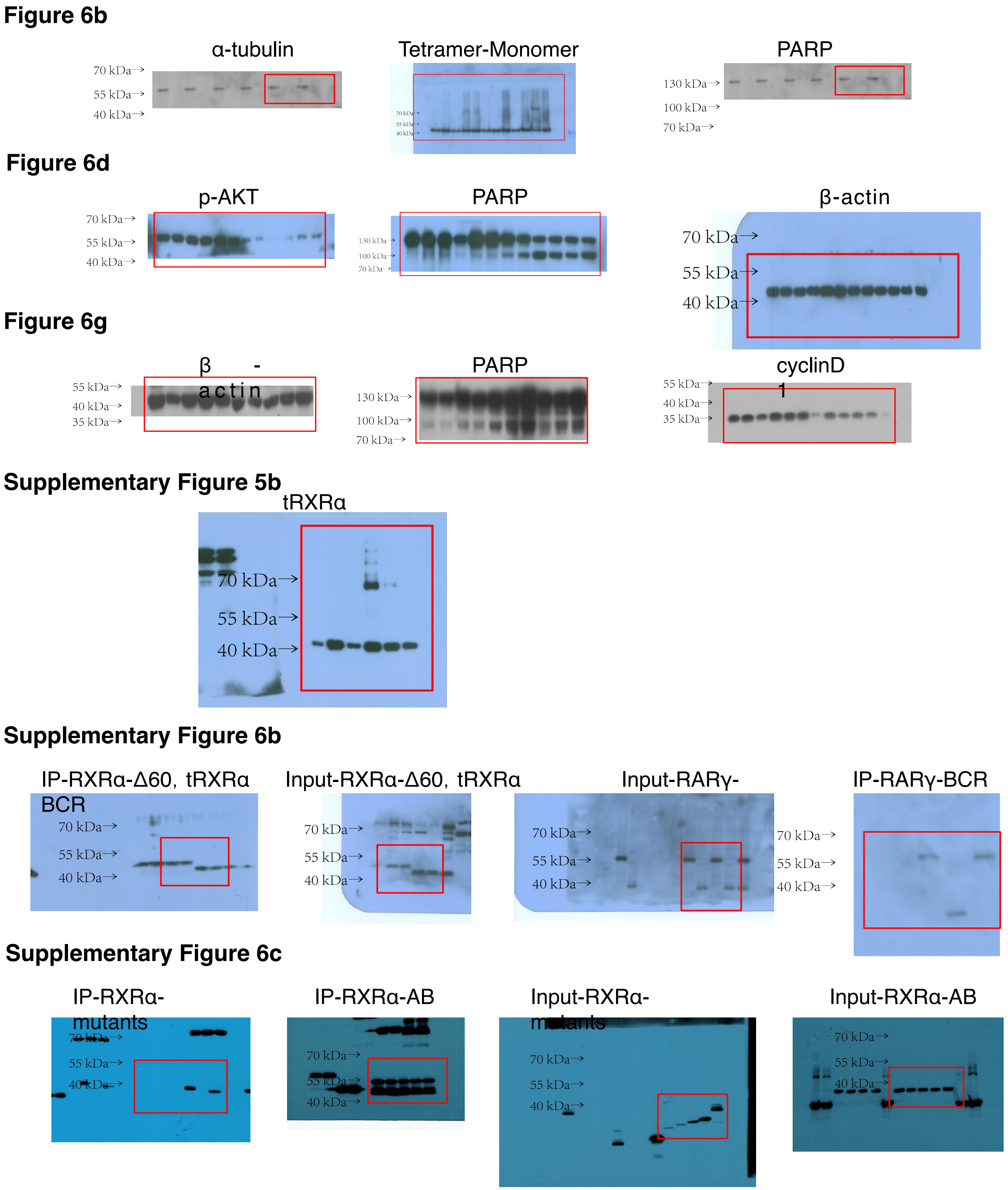

Supplementary Figure 13. Uncropped Western blot images for Figure 6 and Supplementary Figures 5 and 6.

Supplementary Figure 7a

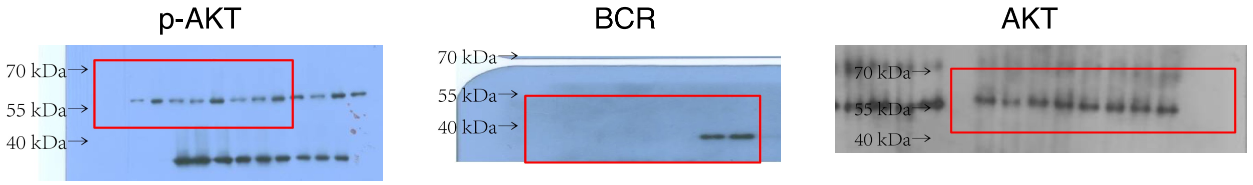

Supplementary Figure 7b

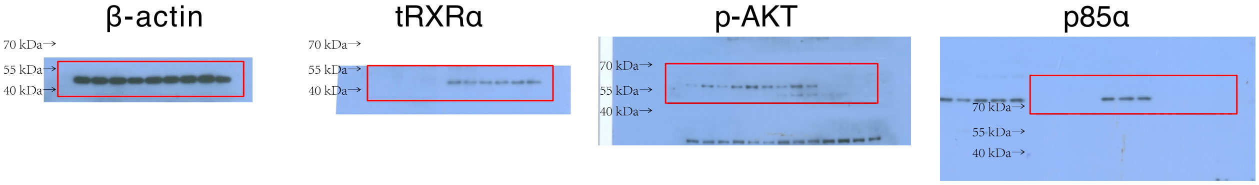

Supplementary Figure 8a

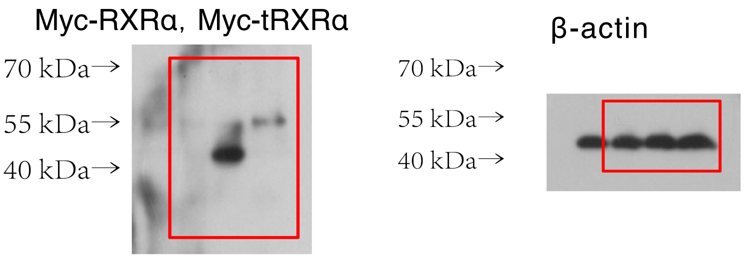

Supplementary Figure 8b

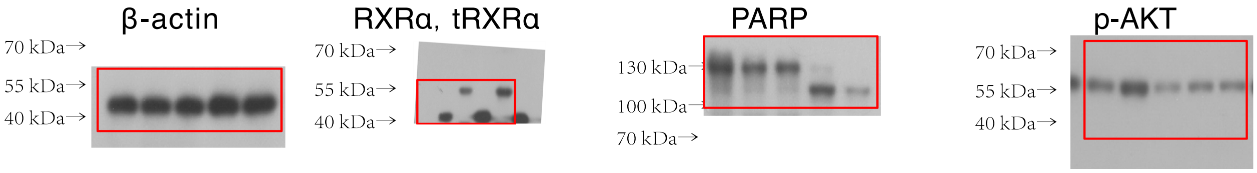

Supplementary Figure 9a

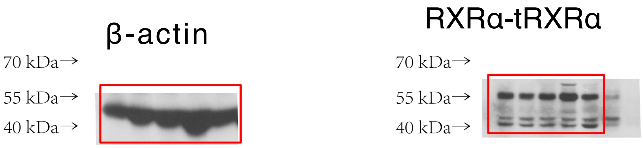

Supplementary Figure 14. Uncropped Western blot images for Supplementary Figures 7, 8, and 9.

Supplementary Table 1. Primers used for constructing expression plasmids

Plasmid	Forward	Reverse
**Myc-RXRα**	CCGGAATTCGGATGGACACCAAACATTTCCTGC	CCGCTCGAGCTAAGTCATTTGGTGCGGCGCCTC
**Myc-RXRα-Δ40**	CCGGAATTCGGATCGGCATCGGCTCCCCGGGACAGC	CCGCTCGAGCTAAGTCATTTGGTGCGGCGCCTC
**Myc-RXRα-Δ60**	CCGGAATTCGGATGGGCCCGCCTTTCTCG	CCGCTCGAGCTAAGTCATTTGGTGCGGCGCCTC
**Myc-tRXRα**	CCGGAATTCGGACCACACCCACCCTGGGC	CCGCTCGAGCTAAGTCATTTGGTGCGGCGCCTC
**Myc-RXRα-Δ100**	CCGGAATTCGGGTCAGCAGCAGCGAGGAC	CCGCTCGAGCTAAGTCATTTGGTGCGGCGCCTC
**Myc-RXRα-ΔAF2**	CCGGAATTCGGATGGACACCAAACATTTCCTGC	CCGCTCGAGGTCAATGGGTGTGTCCCCGATG
**Myc-RXRα-ΔAB**	CCGGAATTCGGTCTGCGCCATCTGCGGGGA	CCGCTCGAGCTAAGTCATTTGGTGCGGCGCCTC
**Myc-RXRα-ΔABΔAF2**	CCGGAATTCGGTCTGCGCCATCTGCGGGGA	CCGCTCGAGGTCAATGGGTGTGTCCCCGATG
**Myc-RXRα-LBD**	CCGGAATTCGGAGCAGCGCCAACGAGGACATGCCGGT	CCGCTCGAGCTAAGTCATTTGGTGCGGCGCCTC
**Myc-tRXRα-ΔAF2**	CCGGAATTCGGACCACACCCACCCTGGGC	CCGCTCGAGGTCAATGGGTGTGTCCCCGATG
**Myc-RXRα-1-235**	CCGGAATTCGGATGGACACCAAACATTTCCTGC	CCGCTCGAGCTAGATCCTCTCCACCGGCATGTCCTC
**Myc-RXRα-AB**	CCGGAATTCGGATGGACACCAAACATTTCCTGC	CCGCTCGAGCTAGATGTGCTTGGTGAAGGAAGCCATG
**Myc-RXRα-LBD/W305Q**	TGCGGGCAGGCCAGAATGAGCTGCTCAT	ATGAGCAGCTCATTCTGGCCTGCCCGCA
**Myc-tRXRα/R316E**	CCTTCTCCCACGAGTCCATCGCCGTGAA	TTCACGGCGATGGACTCGTGGGAGAAGG
**Myc-tRXRα/F313A**	CTCATCGCCTCCGCCTCCCACCGCTCCA	TGGAGCGGTGGGAGGCGGAGGCGATGAG
**HA-RXRα-AB**	CCGGAATTCGGATGGACACCAAACATTTCCTGC	CCGCTCGAGCTAGATGTGCTTGGTGAAGGAAGCCATG
**HA-RARγ**	CCGGAATTCGGATGGCCACCAATAAGGAG	CCGCTCGAGTCAGGCTGGGGACTTCAG
**HA-p85α**	CCGGAATTCGGATGAGTGCTGAGGGGTACCAGTAC	ATAGTTTAGCGGCCGCTCATCGCCTCTGCTGTGCATATAC
**HA-p85α-NIC**	CCGGAATTCGGAGCTCTGATAATACTGAAAACCTCA	ATAGTTTAGCGGCCGCTCATCGCCTCTGCTGTGCATATAC
**HA-p85α-SH3**	CCGGAATTCGGATGAGTGCTGAGGGGTACCAGTAC	CTCGAGCCTTCCAATATATTCTACGTA
**HA-p85α-BCR**	CCGGAATTCGGAAAAAAAATCTCGCCTCCCACACCAA	ATAGTTTAGCGGCCGCTCAGGCTGCTGAGAATCTGAAAAGCATAG
**Flag-p85α-BCR**	CCGGAATTCGGAAAAAAAATCTCGCCTCCCACACCAA	CGGGATCCTCAGGCTGCTGAGAATCTGAAAAGCATAG
**Flag-p85α-BCR-LxxAA**	AGAATTACGACAGGCTGCTGATTGTGATACACC	GGTGTATCACAATCAGCAGCCTGTCGTAATTCT
**His-RXRα-LBD**	GGAATTCCATATGAGCAGCGCCAACGAGGACATGCCGGT	CCGCTCGAGCTAAGTCATTTGGTGCGGCGCCTC
**His-RXRα-LBD/L433D**	GGCTCAAATGCGACGAACATCTCTTCTT	AAGAAGAGATGTTCGTCGCATTTGAGCC
**His-RXRα-LBD/R316E**	CCTTCTCCCACGAGTCCATCGCCGTGAA	TTCACGGCGATGGACTCGTGGGAGAAGG
**His-RXRα-LBD/Q275E**	AGCAGCCGACAAAGAGCTTTTCACCCTG	CAGGGTGAAAAGCTCTTTGTCGGCTGCT
**His-RXRα-LBD/F439A**	ACATCTCTTCTTCGCCAAGCTCATCGGGG	CCCCGATGAGCTTGGCGAAGAAGAGATGT
